# Adaptation and validation for Colombia of the WHO safe childbirth checklist

**DOI:** 10.25100/cm.v49i2.2710

**Published:** 2018-09-30

**Authors:** Ana Carolina Amaya-Arias, Oscar Zuluaga, Douglas Idárraga, Javier H Eslava-Schmalbach

**Affiliations:** 1 Centro de Desarrollo Tecnológico - Sociedad Colombiana de Anestesiología y Reanimación (S.C.A.R.E). Bogota, Colombia.; 2 Hospital Universitario Nacional de Colombia, Facultad de Medicina- Universidad Nacional de Colombia. Bogota, Colombia.

**Keywords:** Maternal mortality, perinatal mortality, patient safety, checklist, World Health Organization, parturition, delivery, obstetric, Morbi-mortalidad materna, morbi-mortalidad neonatal, seguridad del paciente, listas de verificación, Organizacion Mundial de la Salud, parto, obstétrico

## Abstract

**Introduction:**

Most maternal that deaths occur in developing countries are considered unfair and can be avoided. In 2008, The WHO proposed a checklist for delivery care, in order to assess whether a simple, low-cost intervention had an impact on maternal and neonatal mortality in low-income countries.

**Aim:**

To translate, adapt and validate the content of the WHO Safe Childbirth Checklist (SCC) for its use in Colombia

**Methods:**

The translation of the list was carried out, adaptation was made to our context and validation of content through a panel of experts composed of 17 health workers with experience in maternal and neonatal care and safety. The reliability among the judges was calculated (Rwg) and according to the results, items were modified or added to each section of the list.

**Results:**

Modifications were made to the wording of 28 items, none was eliminated, and 19 new items were added. The most important modifications were made to the management guidelines that accompany each item and the items included refer to risks inherent to our environment.

**Conclusion:**

The Colombian version of the SCC will be a useful tool to improve maternal and neonatal care and thereby will contribute to reducing maternal and neonatal morbidity and mortality in our country.

## Resumen

**Introducción:**

La mayor parte de las muertes maternas ocurren en países en vías de desarrollo, se consideran injustas y pueden ser evitadas. En el 2008 la OMS propuso una lista de verificación para la atención del parto, con el fin de evaluar si una intervención simple, de bajo costo, tenía impacto sobre la mortalidad materna y neonatal en los países de bajos ingresos.

**Objetivo:**

Traducir, adaptar y validar el contenido de la lista de verificación del parto seguro de la OMS para su uso en Colombia

**Métodos:**

Se realizó la traducción de la lista, se realizó la adaptación a nuestro contexto y validación de contenido a través de un panel de expertos compuesto por 17 trabajadores de la salud con experiencia en seguridad y atención materna o neonatal. Se calculó la fiabilidad entre los jueces (Rwg) y de acuerdo a los resultados se modificaron o agregaron ítems a cada apartado de la lista.

**Resultados:**

Se hicieron modificaciones a la redacción de 28 ítems, no se eliminó ninguno, y se agregaron 19 nuevos ítems. La mayor parte de modificaciones importantes se hicieron a las orientaciones de manejo que acompañan cada ítem y los ítems incluidos se refieren a riesgos propios de nuestro medio.

**Conclusión:**

La versión para Colombia de la Lista de Verificación del Parto Seguro será una herramienta útil para mejorar la atención a las maternas y neonatos y con esto contribuir a recudir la morbi-mortalidad materna y neonatal en nuestro país.

## Introduction

Reducing maternal mortality is a public health priority worldwide and is one of the "Millennium Development Goals" of the United Nations ^1^. According to figures from the World Health Organization (WHO), approximately 350,000 maternal deaths occur each year ^2^ ^,^ ^3^; most of these deaths occur in underdeveloped or developing countries, and most of them are considered unfair and can be avoided through timely interventions based on evidence ^4^,^5^. In consequence, the Sustainable Development Agenda agreed as Goal 3.1 to reduce the global maternal mortality ratio (MMR) to less than 70 per 100,000 live births between 2016 and 2030 ^6^.

In Colombia, in 2014, the Ministry of Health and Social Protection estimated, based on DANE figures, an MMR of 53.7 per 100,000 live births; however, indicators by department show great inequities in the country, being the departments with higher poverty indexes those that have the highest maternal MMR, surpassing the national indicator even by three or four times ^7^. Faced with this scenario, it is necessary to redouble efforts to achieve the commitments made, especially in the regions with the greatest inequities in terms of health in the country.

Interventions based on checklists have become more frequent in recent years to assist the management of complex or neglected tasks that are at risk of more mistakes. These are lists of items to be checked during a complex activity, as a memory aid, to ensure the correct execution of a task. Integration of checklist programs into clinical practice has shown to reduce mortality and the incidence of complications in surgery and intensive care ^8^ ^-^ ^11^.

A strategy based on a checklist is well adapted to childbirth care if it considers several characteristics of the event, for example, that the main causes of maternal and perinatal mortality are well described; that most deaths occur within a short period of time (twenty-four hours after birth); that international guidelines for best practices exist but are not followed; and that some proven interventions are relatively inexpensive, cost-efficient, easy to implement, but may be difficult to remember and to implement in the appropriate sequence, which could be solved by using a checklist ^12^ ^,^ ^13^.

For this reason, in 2008 the WHO established a safety program based on the Safe Childbirth Checklist to determine whether a simple, low-cost intervention had an impact on maternal and neonatal mortality in low-income countries. The initially proposed checklist contains 29 items addressing the leading global causes of maternal death (hemorrhage, infection, and hypertensive disorders); childbirth-related fetal death (inappropriate intrapartum care); and neonatal death (birth asphyxia, infection, and complications related to prematurity). It also addresses childbirth care (through both caesarean section and vaginal delivery), and simultaneously controls all preventable direct and indirect causes of maternal mortality within the first 24 hours after birth until discharge from hospital.

Pilot test results showed that the implementation of the childbirth checklist led to improved quality of care provided by health workers who attend institutional births ^14^, in addition to being inexpensive, easy to carry out and with evidence of good results in 10 countries of Africa and Asia ^5^.

Due to these results, in 2012, the WHO undertook an initiative that involved the collaboration of 29 countries and 34 research groups that mainly sought to evaluate the factors that facilitated or hindered the application and effective use of this instrument, leading to the design of an implementation guide and an updated checklist ^15^. However, it has not yet been implemented in Colombia, and studies are needed to determine its applicability and acceptance in our context.

In order to use this instrument with a different population, its format should be modified to suit the context in which it will be used. Therefore, within the framework of the worldwide pilot study, the checklist had to be translated and adapted to our context. This study sought to translate, adapt and validate the content of the WHO Safe Childbirth Checklist for its use in Colombia.

## Materials and Methods

### Design

Validation study of instruments.

### Selection and description of participants

A team of 17 experts in patient safety, obstetrics, maternal and neonatal safety and mortality were available for expert consensus: 6 anesthesiologists, 1 internist, 5 obstetrician-gynecologists, 4 nurses and 1 pediatrician neonatologist. Most of them had more than 10 years of experience, and only 2 had less than that (7 and 2 years); they were selected by expert referral. The list of expert judges can be seen in Annex 1.

### Process

The indications of the American Psychological Association (A.P.A.) for the adaptation of measuring instruments were followed to adapt the instrument, which include: 1) making sure that there is equivalence between languages and cultural groups of interest, 2) deciding whether to adapt an existing instrument or prepare a new one, 3) selecting qualified professional translators, 4) performing direct or inverse translation, 5) reviewing the adapted version of the instrument and making the necessary corrections, among others ^16^ ^,^ ^17^. With these recommendations in mind, this study followed the steps below:

#### Phase 1. Translation

The original instrument was translated by a qualified Colombian translator; this version was reviewed by the co-researchers of the project. The final adjustments to the translation were made in accordance with their suggestions and observations.

#### Phase 2. Content validity

The evaluation of the contents is usually performed by a panel of experts or expert judges, and is defined as "an informed opinion of people with experience in the subject, who are recognized by others as qualified experts, and who can provide information, evidence, judgments and assessments ^18^.” In this study, content validity was analyzed by means of a consensus of evaluators applying the discussion and consensus methodology among the experts from a modified Delphi ^19^.

The group of judges received a format to assess the clarity and relevance of each item in the checklist, as well as their sufficiency to measure each phase of childbirth care proposed in the original instrument. The scores given to each behavior or section, in each of these categories, ranged from 1 to 4 on a Likert scale, according to the criteria shown in Table 1.

**Table 1:** Definition of each category and scores to be applied in the valuation of the items and sections of the list.

**Clarity (Cla)**
The item is easily understood, i.e. its syntax and semantics are appropriate
1. The item is not clear
2. The item requires several modifications or a very large modification regarding the use of the words according to their meaning or order.
3. A very specific modification of some of the terms of the item is required.
4. The item is clear and has appropriate semantics and syntax
**Relevance (Rel)**
The item you are reviewing is important for the application of the checklist as it relates to an important and necessary aspect that has to be checked during labor and should therefore be included in the instrument.
1. The item can be removed.
2. The item has some relevance, but another item may include what it measures.
3. The item is important but is not decisive enough to be included.
4. The item is very important, it should be included.
**Sufficiency (Suff)**
The items related to the same moment in the checklist are sufficient to cover the essential aspects that need to be verified during labor.
1. The items are not sufficient.
2. Items cover some part, but not all of it; items should be added or changed.
3. Few items should be added or changed in order to cover the essential aspects to be verified during labor.
4. The items are sufficient to obtain a complete measurement of the essential aspects to be verified during labor.

Reliability between judges (𝒓_𝑾𝑮_ *: within-group interrater reliability*) was calculated for each behavior. This is used to determine the level of agreement among evaluators when validating the items, and is estimated by:

$$r_{WG}\;=\;1\;-\left(\frac{S_{Xj}^{2}}{\sigma_{EU}^{2}}\right)$$

Where 𝒓_𝑾𝑮_ indicates reliability between evaluators, in a group of *k* evaluators per item 

$X_{j}:\;S_{Xj}^{2}$ is the observed variance of X_j_ and $X_{j}\;y\;\sigma_{EU}^{2}$ is the randomly expected variance of X_j_, that is, a condition of reliability between evaluators equal to zero ^20^.

According to this calculation, it possible to decide if an item had to be removed, modified or not, and if new items had to be added or not to each phase of the list. The accepted criterion was that if the level of agreement (Rwg) did not exceed a cut-off point of 0.8, the item or instrument had to be modified ^21^. In this case, Rwg less than 0.8 in clarity indicated the need to modify the item; Rwg less than 0.8 in relevance required discussing whether or not to remove the item; and Rwg less than 0.8 in sufficiency revealed that there were items that needed to be included in the version for Colombia. This process was carried out several times until the modifications made showed agreement among the judges above the cut-off point.

### Instrument

The Safe Childbirth Checklist (SCC) was developed by the WHO along with nurses, midwives, obstetricians, pediatricians, patient safety experts and patients from around the world as a strategy to help health workers improve their practices in maternal and newborn care. It focuses on the leading causes of maternal mortality worldwide (hemorrhage, infection, hypertensive disorder and dysfunctional labor), intrapartum-related stillbirths (inadequate care) and neonatal deaths (events during labor, infections and preterm complications). Each item on the list is a critical action and its omission can lead to serious outcomes.

Pilot tests have shown that the SSC (version 1.0) has improved the practices of health workers ^5^. Four sections (pause points) are proposed and a set of essential practices should be completed (list of items) during each pause. A checklist should be used for each mother and her baby, and each item should be checked by marking it when it has been completed or performed. Nurses, midwives, doctors, or other health care workers are responsible for filling out the form.

Pause points occur during critical situations when complications can be avoided or adequately managed. They also occur at times when it is convenient to check the mother and the newborn. Thus, the Safe Birth Checklist is designed to be used at these four pause points during institutional births:

1. On admission (8 items)
2. Just before pushing or before cesarean section (5 items)
3. Soon after birth within 1 hour (9 items)
4. And, before discharge (7 items)

### Ethical Considerations

This work complies with national and international recommendations for biomedical research ^22^ ^,^ ^23^. The research protocol was approved by the Ethics Committee of the Universidad Nacional de Colombia.

### Bias Control

In order to control possible bias among members of the consensus, a modified Delphi was applied, for which the experts gave their scores individually and blinded to the scores of the other experts first. Then each expert received a scoring device on the day of face-to-face consensus, so that each one gave again an individual score, blinded to the scores of the other experts.

## Results

**Figure 1:**
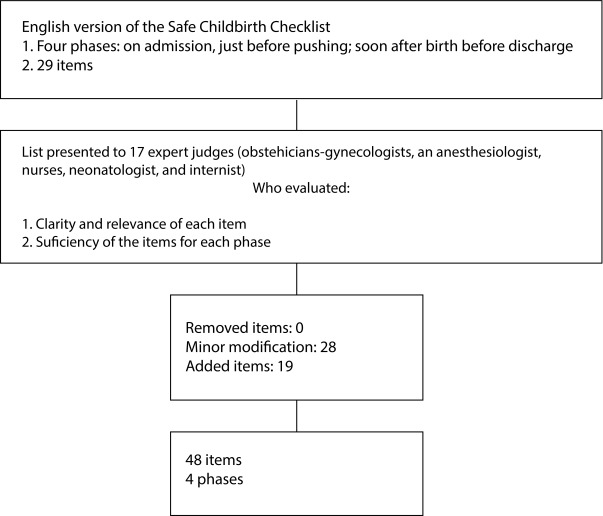
Flowchart. Content Validation of the Safe Birth Checklist for Colombia.

Once reliability among judges (Rwg) for each item and care phase was established, and taking into account the decision criteria, modifications were made to 28 items, in order to make them more suitable for use in our context. No items were removed, and 1 item remained the same; the results are presented in Figure 1 and Table 2.

**Table 2:** Percentage of agreement on the categories "clarity and coherence" and final decision on each item

No	Initial item		Clarity rWG	Relevance rWG	Decision	Final item
Before the Birth							
1	Does the mother need referral?	Check your facility’s criteria	0.71	1.00	Modify	Does the mother need referral?	Check your facility’s criteria
	❏ No					❏ No	
	❏ Yes, organized				Do not remove	❏ Yes, the corresponding procedures were organized	
2	Partogram started?	Start plotting when cervix is ≥4 cm	0.62	1.00	Modify	Partogram started?	Start plotting when cervix is ≥4 cm
	❏ No: will start when 4≥cm	then the cervix should dilate ≥1 cm/h				❏ No: will start when cervical dilation is ≥4cm	● Every 30 minutes-plot heart rate and fetal heart rate (FHR). During expulsion, FHR should be plotted every 10 min
	❏ Yes	● Every 30 min.: Plot HR, contractions, FHR				❏ Yes	● Every hour plot: vital signs and uterine activity
		● Every 2 hours: Plot temperature			Do not remove		● Every 2 h: Plot blood pressure
		● Every 4 Hours: Plot Blood Pressure					● Every 4 h: Plot temperature
3	Antibiotics?	Give antibiotics to the mother if any of:	0.45	0.86	Modify	Antibiotics?	Give antibiotics to the mother if any of:
	❏ No	● Temperature ≥38° C				❏ No	● Temperature ≥38º C
	❏ Yes, given	● Foul-smelling vaginal discharge				❏ Yes, given	● Foul-smelling vaginal discharge
		● Rupture of membranes >18 h			Do not remove		● Confirmed carrier of *Streptococcus Agalactiae*
							● Unknown carrier of *Streptococcus Agalactiae* with gestational age less than 37 weeks or rupture of membranes greater than 18 h
							● Clinical suspicion of infection in any site
4	Magnesium Sulfate?	Give magnesium sulfate to mother if any of:	0.44	0.88	Modify	Magnesium Sulfate?	Give magnesium sulfate to mother if any of:
	❏ No	● Diastolic BP ≥110 mmHg and 3+ proteinuria				❏ No	● Systolic blood pressure ≥140 or diastolic blood pressure ≥90, accompanied by any of the following: Severe headache, visual alterations such as visual disturbance or phosphenes, pain in right hypochondrium or epigastric pain, vomiting, hematuria, oliguria, thrombocytopenia or suspected Hellp syndrome.
	❏ Yes, given	● Diastolic BP ≥90 mmHg, 2+ proteinuria and any: severe headache, visual disturbance, epigastric pain.				❏ Yes, given	● Systolic blood pressure ≥160 or diastolic blood pressure ≥110
					Do not remove		● Eclampsia (Seizures)
							● Gestational age ≤32 weeks with viable fetus and active labor (fetal neuroprotection).
5	Antiretrovirals?	Mothers with CD4 ≤350 or clinically diagnosed with the virus require treatment. Mothers with CD4 >350 require prophylaxis	0.52	0.92	Modify	Antiretrovirals?	In case of positive HIV test, administer antiretrovirals following the institutional protocol. If the status is unknown, order a rapid HIV test and define management according to the result and following the institutional protocol.
	❏ No, HIV negative is confirmed					❏ No, HIV negative is confirmed	
	❏ Yes, given				Do not remove	❏ Yes, given	
	If status is unknown, order HIV testing						
6	Confirm availability of hand asepsis supplies and use of gloves for each vaginal exam.		0.62	1.00	Modify	Confirm availability of supplies and compliance with hand washing protocol.	Use sterile gloves in case of rupture of membranes.
					Do not remove	Confirm availability and use of gloves for each vaginal exam	
7	Encourage the presence of a companion at the time of delivery.		0.48	0.88	Modify	The presence of a companion during labor was encouraged and allowed	
					Do not remove		
8	Confirm that the mother/accompanying person will ask for help during labor, in case of:	● Bleeding	0.32	0.96	Modify	The pregnant woman or companion recognizes the warning signs and will ask for help in case of: bleeding, paleness, sweating, permanent abdominal pain, severe headache, visual or hearing disturbances, trouble urinating, pushing sensation	
		● Severe abdominal pain					
		● Severe headache or visual problems					
		● Trouble urinating					
		● Need to push			Do not remove		
**Before Pushing (Or Before Caesarean)**							
9	Does mother need to start:	Give antibiotics to mother if any of:	0.36	0.92	Modify Do not remove	The mother requires:	Give antibiotics to mother if any of:
	Antibiotics?	● Temperature ≥38° C				Antibiotics?	● Temperature ≥38º C
	❏ No	● Foul-smelling vaginal discharge				❏ No	● Foul-smelling vaginal discharge
	❏ Yes, given	● Rupture of membrane>18 h				❏ Yes, given	● Unknown or confirmed carrier of *Streptococcus Agalactiae*
		● Caesarean section					● Clinical suspicion of infection on any site
							● Cesarean section
							● Delivery under unsafe conditions
10	Magnesium sulfate?	Give magnesium sulfate to mother if any of:	0.38	0.92	Modify	Magnesium sulfate?	Give magnesium sulfate to mother if any of:
	❏ No	● Diastolic BP ≥110 mmHg and 3+ proteinuria				❏ No	● Systolic blood pressure ≥140 or diastolic blood pressure ≥90, accompanied by any of the following: Severe headache, visual disturbances such as blurred vision or phosphenes, pain in right hypochondrium or epigastric pain, vomiting, hematuria, oliguria, thrombocytopenia or suspected Hellp syndrome.
	❏ Yes, given	● Diastolic BP ≥90 mmHg, 2+ and any: severe headache, visual disturbance, epigastric pain.				❏ Yes, given	● Systolic blood pressure ≥160 or diastolic blood pressure ≥110
					Do not remove		● Eclampsia (Seizures)
							● In patients with severe pre-eclampsia, continue magnesium sulphate for up to 24 h after delivery.
11	For mother	Prepare to care for mother immediately after birth:	0.27	0.96	Modify	For mother	Prepare to care for mother immediately after birth:
	Gloves	Confirm single baby only (not multiple birth).				Sterile gloves	Confirm single baby only (not multiple birth).
	Alcohol-based hand rub or soap and clean water	1. Give oxytocin within 1 minute after birth.				Antiseptics for hand washing and patient preparation according to institutional protocol	● Perform active management of the third stage according to institutional protocol.
	Oxytocin 10 units in syringe	2. Deliver placenta			Do not remove	Uterotonics, preferably Oxytocin	● Confirm uterus is contracted.
		3. Confirm uterus is contracted.					
12	For baby	Prepare to care for baby immediately after birth:	0.41	1.00	Modify	For the newborn	Immediate care for the newborn:
	Clean towel	1. Dry baby, keep warm				Clean towel	● Dry the baby and keep it warm.
	Sterile blade to cut cord	2. If not breathing, stimulate and clear airway				Sterile blade to cut cord	If not breathing, stimulate and clear airway.
	Suction device	3. If still not breathing:				Rubber tie, plastic clip or sterile umbilical tapes	● If still not breathing:
	Bag-and-mask	● Clamp and cut cord			Do not remove	Heat source	● Clamp and cut cord
		● Ventilate with bag-and-mask				Suction device	● Ventilate with bag-and-mask
		● Shout for help				Bag-and-mask	● Shout for help
							● Follow institutional protocol for neonatal resuscitation
13	Assistant identified and ready to help at birth if needed?		0.51	0.57	Modify After deliberation it was decided	If necessary, an assistant (health personnel/accompanying person) is available to assist in delivery care.	
					not to remove it		
**Soon After Birth (within 1 hour)**							
14	Is mother bleeding abnormally?	If bleeding abnormally:	0.45	1.00	Modify	Is mother bleeding abnormally?	If bleeding abnormally:
	❏ No	● Massage uterus				❏ No	● Hemoclassification
	❏ Yes, shout for help	● Consider more uterotonic				❏ Yes, active institutional protocol for obstetric hemorrhage management	● Massage uterus
		● Start IV fluids			Do not remove		● Consider more uterotonic
		● Treat cause: uterine atony, retained placenta/fragments, vaginal tear, uterine rupture					● Initiate IV fluids and consider blood products
							● Treat cause: tone, trauma, tissue, thrombin (Four Ts mnemonic), uterine atony, retention of placental debris, vaginal tear, uterine rupture, coagulopathy
15	Does mother need to start:	Give antibiotics to mother if placenta manually removed or if mother’s temperature ≥38° C and any of:	0.56	0.92	Modify	Does mother need to start:	Give antibiotics to mother if any of:
	Antibiotics?	● Chills				Antibiotics?	● Instrumented childbirth
	❏ No	● Foul-smelling vaginal discharge				❏ No	· Manual removal of placenta.
	❏ Yes, given					❏ Yes, given	● Uterine revision
					Do not remove		● Severe perineal tear
							● Temperature ≥ 38º C
							● Foul-smelling vaginal discharge
							● Clinical suspicion of infection in any location
16	Magnesium sulfate?	Give magnesium sulfate to mother if any of:	0.62	0.60	Modify After deliberation it was decided	Magnesium sulfate?	Give magnesium sulfate to mother if any of:
	❏ No	● Diastolic BP ≥110 mmHg and 3+ proteinuria				❏ No	● Systolic blood pressure ≥140 or diastolic blood pressure greater than or equal to 90, accompanied by any of the following: Severe headache, visual disturbances such as blurred vision or phosphenes, dolor en right or epigastric hypochondrium, vomiting, hematuria, oliguria, thrombocytopenia or suspected Hellp syndrome.
	❏ Yes, given	● Diastolic BP ≥90 mmHg, 2+ proteinuria, and any: severe headache, visual disturbance, epigastric pain.				❏ Yes, given	● Systolic blood pressure ≥160 or diastolic blood pressure ≥110
					not to remove it		· Eclampsia (Seizures)
							● Administer magnesium for 24 h in a patient with severe pre-eclampsia.
17	Does baby need:	Check your facility’s criteria	0.84	>1.00	Do not modify	Does baby need:	Check your facility’s criteria
	Referral?					Referral?	
	❏ No				Do not remove	❏ No	
	❏ Yes, organized					❏ Yes, organized	
18	Antibiotics?	Give baby antibiotics if antibiotics given to mother for treatment of maternal infection during childbirth or if baby has any of:	0.62	0.96	Modify	Antibiotics?	Give antibiotics to the newborn if mother has been given antibiotics for obstetric infection or if any:
	❏ No					❏ No	● Respiratory rate >60/min
	❏ Yes, given	● Respiratory rate >60/min or <30/min				❏ Yes, given	● Thoracic retraction or rales
		● Chest in-drawing, grunting, or convulsions					● Poor response to stimulus
		● Poor movement on stimulation					● Temperature <35º C (and not rising after warming), or temperature ≥38º C
		● Baby’s temperature <35° C (and not rising after warming) or baby’s temperature ≥38° C			Do not remove		● Suspicion of congenital syphilis
							● APGAR less than 7
							Note: Antibiotics should NOT be given to patients with HR <30 min or seizures.
			
19	Special care and monitoring?	Arrange special care/monitoring for baby if any:	0.72	1.00	Modify	Special care and monitoring?	Arrange special care/monitoring for baby if any:
	❏ No	● More than 1 month early				❏ No	● Gestational age <37 weeks
	❏ Yes, organized	● Birth weight <2,500 g			Do not remove	❏ Yes, organized	● Birth weight <2,500 g
		● Needs antibiotics					● Needs antibiotics
		● Required resuscitation					● Required neonatal resuscitation
20	Antiretrovirals?	If the mother is HIV+, follow the institution's local guidelines for the baby (prophylaxis should begin within 12 h after delivery).	0.71	0.96	Modify	Antiretrovirals?	If HIV-positive, administer antiretrovirals according to institutional protocol for the newborn (prophylaxis should begin within 12 h after delivery)
	❏ No					❏ No	
	❏ Yes, organized				Do not remove	❏ Yes, given	
21	Started breastfeeding and skin-to-skin contact (if mother and baby are well).		0.72	1.00	Modify Do not remove	Started breastfeeding and skin-to-skin contact (if mother and baby are well).	
22	Confirm mother / companion will call for help if danger signs present.		0.59	0.55	After deliberation it was decided not to remove it	The mother or companion recognize warning signs and will ask for help in the event of:	
						Mother: Bleeding, paleness, sweating; permanent abdominal pain, severe headache, visual or hearing problems, trouble urinating, pushing sensation.	
						Baby: difficulty breathing/fast breathing, fever, cold skin, weak sucking, drowsiness, or difficulty waking up.	
**Before Discharge**							
23	Is mother bleeding abnormally?		0.53	0.62	Modify After deliberation it was decided	Is mother bleeding abnormally?	If bleeding is abnormal:
	❏ Yes: treat and delay discharge					❏ Yes: treat and delay discharge	● Massage uterus
	❏ No					❏ No	● Consider more uterotonic
					not to remove it		● Initiate IV fluids and consider blood products.
							● Treat cause: tone, trauma, tissue, thrombin (Four Ts mnemonic), uterine atony, retention of placental debris, vaginal tear, uterine rupture, coagulopathy
24	Does mother need to start antibiotics?	Give antibiotics to mother if mother’s temperature ≥38° C and any of:	0.62	0.92	Modify Do not remove	Does mother need to start antibiotics?	Give antibiotics to mother if any of:
	❏ No					❏ No	● Mother’s temperature ≥38° C in the last 24 h
	❏ Yes, given and delay discharge					❏ Yes, given and delay discharge	● Foul-smelling genital bleeding
		● Chills					● Clinical suspicion of infection in any location
		● Foul-smelling vaginal discharge					● Signs of superficial or deep surgical site infection (episyorrhaphy, surgical wound, endometritis, myometritis, etc.)
25	Does baby need to start antibiotics?	Give antibiotics to baby if any of:	0.71	1.00	Modify Do not remove	Does baby need to start antibiotics?	Give antibiotics to baby if any of:
	❏ No	● Respiratory rate >60/min or <30/min				❏ No	● Respiratory rate >60/min or <30/min
	❏ Yes, give antibiotics, delay discharge, give special care.	● Chest in-drawing, grunting, or convulsions				❏ Yes, give antibiotics, delay discharge, give special care and monitor.	● Chest in-drawing, grunting, or convulsions
		● Poor movement on stimulation					● Poor movement on stimulation
		● Baby’s temperature <35° C (and not rising after warming) or baby’s temperature ≥38° C					● Baby’s temperature <35° C (and not rising after warming) or baby’s temperature ≥38° C
		● Stopped breastfeeding well					● Stopped breastfeeding well
		● Umbilicus redness extending to skin or draining pus					● Umbilicus redness or draining pus
26	Is baby feeding well?		0.71	1.00	Modify Do not remove	Is baby feeding well?	
	❏ No: establish good breastfeeding practices and delay discharge					❏ No: delay discharge until good breastfeeding practices are established	
	❏ Yes					❏ Yes	
27	If mother is HIV-positive, do both mother and baby have antiretroviral treatment (ARVs) for 6 weeks?		0.62	0.92	Modify Do not remove	If the mother is HIV-positive, do both mother and baby have antiretroviral treatment (ARVs) for 6 weeks?	If mother is HIV-positive:
	❏ Yes					❏ Yes	● Schedule appointment with infectology or outpatient consultation with HIV program for both the mother and baby within one month
						❏ No, suspend breastfeeding and follow institutional protocol	
28	Discuss and offer family planning options to mother		0.73	0.95	Modify Do not remove	Family planning counseling and referral for administration of selected method provided	
29	Arrange follow-up and confirm mother / companion will seek help if danger signs appear after discharge.		0.78	1.00	Modify Do not remove	The mother or companion recognize warning signs and will seek help if:	
						Mother: Bleeding, severe abdominal pain, severe headache, visual disturbance, shortness of breath, fever or chills, difficulty urinating.	
						Baby: difficulty breathing/fast breathing, fever, cold skin, weak sucking, drowsy or hard to wake up, yellowing of the eyes, skin, palms of hands, or soles of feet.	

As Table 2 shows, the most important modifications were made to the management guidelines that accompany each item and some minor modifications to the wording or the terms used in them.

Furthermore, 19 new items were added: 4 in the admission phase, 9 before delivery, 2 soon after birth and 4 in the discharge phase. The 4 items of the admission phase refer to allergy review, need for antihypertensive and syphilis treatment, while the supplies item was divided into two parts. The 9 items added to the section before birth are explained by the fact that the supplies item was considered as one, while it was separated in the new version; in addition questions about partogram and the need for antihypertensive treatment were added. In the section soon after birth, two items were added regarding the need for an antihypertensive medication for the mother and screening for congenital hypothyroidism and hemoclassification for the newborn. Finally, in the section before discharge, 4 items were added that refer to investigating if anemic syndrome is observed in the mother, if a postpartum control appointment was scheduled, if treatment for syphilis was given, and if catheters and foileys were used.

The final version of the Safe Birth Check List for use in Colombia can be seen in Annex 2

## Discussion

The Safe Childbirth Checklist ^5^, as its surgery counterpart ^8^, is an instrument used to optimize standardized processes followed by health personnel, making sure that clinicians take into account events and actions that each stage of labor may require to provide care to patients with better quality. It is not a guide to clinical practice, yet it provides a minimum standard of care, favoring assessment during each birth and considering basic behaviors relevant to each patient, contributing significantly to clinical safety for the patient and legal security for the staff that provide care. To incorporate these benefits to the maximum, the instrument requires to be adapted to the context in which it will be applied ^14^ ^,^ ^15^, as exposed in this article.

Thus, following the methodology described above, after the translation of the original document and after reviewing and adjusting the Spanish version of each question and its recommendations, consensus points were modified and added to each of the four items on the list. For the first section, admission, all items were modified, adding considerations to each question, and formulating four new ones. Thus, regarding partogram, the suggested times for monitoring signs were modified. The proposal to take blood pressure every 2 hours instead of every 4 hours was considered because every 4 hours is a very long period of time to assess the impact of possible measures that may be taken. Measuring temperature every 4 hours rather than every 2 hours is explained by the fact that fever during labor is considered infrequent and that this parameter is not variable as to look for alterations so frequently. Controlling maternal and fetal heart rate every 30 minutes is also necessary, as well as measuring uterine activity every hour, since these parameters present a rapid variation based on the change of clinical conditions and the interventions in the patient and the neonate.

Regarding the use of antibiotics for the mother, modifications were made to several items on the list. On admission, antibiotic therapy was added in patients confirmed as carriers of *S. Agalactiae* ^24^ in whom there is suspicion of gestation of less than 37 weeks or with a rupture of membranes of 18 hours, because this infection very often leads to early neonatal sepsis, which in many cases leads to neonatal death. It is worth noting that, in case of clinical suspicion of an infection in the mother in any site, the corresponding antibiotic therapy should be initiated, since such infection could complicate labor with unfavorable outcomes for the mother and/or the newborn. With respect to the use of magnesium sulphate, considerations for administration were extended, especially its use in case of eclampsia ^25^.

New questions were also added. One is related to the presence of allergies in the mother, because it is essential to know, from the very beginning of childbirth care, whether the patient may present an allergic reaction that manifests itself as an anaphylactic shock that could lead to death. The second refers to whether the mother requires treatment for syphilis, considering the high prevalence of this disease in our context and the possibility of transmission to the fetus, thus protecting the mother and preventing congenital syphilis. Treatment for syphilis with antitreponemal antibiotic therapy is categorical and determinant if the mother is diagnosed with syphilis ^26^. If the status of syphilis is unknown, the institutional protocol for syphilis test should be followed and treatment should be defined according to the results. The third question asks about whether the mother requires antihypertensive management, taking into account that gestational hypertension could cause maternal and fetal morbidity and mortality; this is a predisposing factor to the development of potentially fatal complications such as placental abruption, brain hemorrhage, hepatic and renal failure and disseminated intravascular coagulation ^27^.

With respect to the second section, just before the expulsion or caesarean section, some questions were added. One refers to the initiation of antihypertensives and another to the evolution of the partogram, because answering these questions seeks to predict complications and the need for interventions at birth. Suspicion or infection by *S. Agalactiae* was added to the section that refers to antibiotic therapy for the mother, and the need to initiate antibiotic therapy if there is a suspicion of infection of any site. The indication for antibiotic therapy was removed when prolonged rupture of membranes occurs (more than 18 hrs) since this consideration should be made on admission; it has no place in this section either if the patient was admitted to the institution during expulsion, because it should be answered at both moments of the checklist, both in the admission section (where this indication is referred) and in the just before childbirth or cesarean section.

During the third section, after delivery or cesarean section (and up to one hour later), the mother is also asked whether she took magnesium sulphate during pregnancy, and the clinician is asked to consider the same indications as in the previous two sections. This part also refers to the use of antibiotic therapy, extending the indications of the original list by adding use of antibiotics for instrumental delivery, manual removal of the placenta, uterine revision, severe perineal tear and clinical suspicion of infection of any site. This section also includes the baby, considering indications of need of referral, antibiotic therapy and special monitoring, which were maintained. Modifications were made to the item antibiotic therapy, ruling out neonatal seizures at this stage as an indication for administering antibiotic therapy because this symptom, right at birth, is considered to be caused by clinical situations other than neonatal infection, such as hydroelectrolytic disorders, hypoglycemia and metabolic disorders. Antibiotic therapy for congenital syphilis and APGAR less than 7 (which predisposes to more infections in neonates) were added. A new question was added regarding screening for congenital hypothyroidism and blood sample collection for hemoclassification in the newborn, which should occur during labor, before expulsion, by collecting blood from the umbilical cord, to avoid unnecessary puncture in the baby and considering that the collection of the sample after one hour leads to false results ^28^. It is important to establish if the baby has hypothyroidism, since this pathology must be treated since birth as it may cause alterations, including neurological development alterations.

Finally, the last section of the checklist, before discharging both the mother and the baby, situations in which antibiotics should be administered were also established. Antibiotic therapy should be given to every mother with temperature above or equal to 38°C and foul-smelling vaginal bleeding, which coincides with the original checklist; however, initiating antibiotic treatment when there are signs of infection in the surgical site, superficial and deep and a clinical suspicion of infection in any site was added. As for the baby, the same indications established in the original checklist were maintained, because, at this point, the probability that neonatal seizures are generated by infection is very high. Regarding maternal HIV infection, the need for follow-up due to infectious diseases was added for both the mother and the baby for long-term control. The moments in which the postpartum check-up appointment should be granted by the outpatient clinic were clarified, so an appointment at seven days is recommended if the delivery was low risk and at 48 hours if there were risk factors. Finally, questions of whether the mother and the baby received syphilis treatment, if the test was positive, and if probes and catheters were removed, were added; this seeks to avoid discharging the patient and her baby with possible infections or risk factors that may lead to an infection.

## Conclusion

The Colombian version of the Safe Childbirth Checklist is expected to become an instrument useful to support institutions and improve care for mothers and newborns, while supporting the fulfillment of the objectives of sustainable development in our country. In addition, we expect that its implementation is especially useful in remote areas, where many safe practices are not systematically followed. All of this is intending to contribute to reducing maternal and neonatal morbidity and mortality.

## Anexo 1 Panel de Expertos

Médicos Especialistas: Alejandro Antonio Bautista Charry. Médico Ginecobstetra. Director departamento obstetricia y ginecología, Universidad Nacional de Colombia. 26 años de experiencia. E-mail: aabautistac@unal.edu.co Alejandro León Upegui Saldarriaga. Médico anestesiólogo. Clínica Universitaria Bolivariana. 18 años de experiencia. E-mail: alus@une.net.co Ana María Guevara Zambrano. Médico Ginecobstetra. Clínica Universitaria Bolivariana, Hospital Pablo Tobón Uribe. 10 años de experiencia. E-mail: anitaguevara4@yahoo.es Catalina María Valencia González. Médico Ginecobstetra. Jefe de la Unidad Fetal de la Clínica Reina Sofía. 14 años de experiencia. E-mail: cvalencia@colsanitas.com Germán Parra Corredor. Médico anestesiólogo, Clínica Colsánitas. 34 años de experiencia. E- mail: germanparra2000@yahoo.com Jairo Enrique Guerrero Giraldo. Médico Ginecobstetra. Jefe del Departamento de Ginecología y Obstetricia del Centro Médico Imbanaco. 10 años de experiencia. E-mail: jairoenrique.guerrero@imbanaco.com.co Javier Hernando Eslava-Schmalbach. Médico anestesiólogo. Vice decano de investigaciones Universidad Nacional de Colombia. 23 años de experiencia. E-mal: jheslavas@unal.edu.co Jorge Andrés Rubio-Romero. Médico Ginecobstetra. Profesor titular de la Universidad Nacional de Colombia. 34 años de experiencia. E-mail: jarubior@unal.edu.co José Antonio Rojas Suarez. Médico anestesiólogo. Coordinador de la Unidad de Cuidados Intensivos, Gestión Salud de Cartagena. 10 años de experiencia. E-mail: joseantonio.rojas.suarez@gmail.com Juan Carlos Bocanegra. Médico anestesiólogo. Clínica Universitaria Colombia. 15 años de experiencia. E-mail: bocanegra67@yahoo.com Luz María Gómez. Médico anestesiólogo. Subdirectora científica S.C.A.R.E. 22 años de experiencia. E-mail: lm.gomez@scare.org.co Nicolás Ramós Rodríguez. Pediatra Neonatólogo. Director Programas de Especialización en Pediatría y Neonatología Universidad del Bosque, Presidente Sociedad Colombiana de Pediatría. 25 años de experiencia. E-mail: bosquepediatria@yahoo.com Mauricio Vasco. Médico anestesiólogo. Coordinador comité de anestesia obstétrica S.C.A.R.E. 15 años de experiencia. E-mail: machuchovasco@yahoo.com
**Enfermeras:**
Claudia Patricia Henao López. Enfermera, Clínica Universitaria Bolivariana. 10 años de experiencia. E-mai: claudia.henao@upb.edu.co Dolly Magnolia González Hoyos. Enfermera Jefe. Docente Universidad de Caldas. 34 años de experiencia. E-mail: dolly.gonzales@ucaldas.edu.co Laura Vanessa Osorio Berrio. Enfermera, Clínica Universitaria Bolivariana. 2 años de experiencia. E-mail: laurav.osorio@upb.edu.co Sandra Milena Raigosa Villa. Enfermera, Clínica Universitaria Bolivariana. 7 años de experiencia. E-mail: sandra.raigosa@upb.edu.co

## Anexo 2. Lista de Verificación del Parto Seguro - Versión Colombia

**Antes del parto:** /LISTA DE VERIFICACIÓN DEL PARTO SEGURO

1. En el Ingreso	
¿La madre necesita ser remitida? ❏ No ❏ Sí, se ha organizado	Verifique los criterios de remisión de su institución
¿Se inició el partograma? ❏ No: iniciará a los ≥4cm de dilatación ❏ Sí	Inicie el trazo cuando la dilatación cervical sea ≥4cm, ● Cada 30 minutos: registre la frecuencia cardiaca, contracciones y frecuencia cardiaca fetal. ● Cada 2 horas: registre la temperatura ● Cada 4 horas: registre la presión arterial
¿La madre es alérgica a algún medicamento? ❏ No ❏ Sí ¿Cuál(es)? _____________________________	
La madre requiere:	
¿Antibióticos? ❏ No ❏ Sí, suministrados	Suministre antibióticos a la madre si se presenta cualquiera de las siguientes: ● Temperatura ≥38ºC ● Flujo vaginal fétido ● Portadora desconocida o confirmada de Estreptococo Agalactiae ● Sospecha clínica de infección de cualquier localización
¿Sulfato de Magnesio? ❏ No ❏ Sí, suministrado	Suministre sulfato de magnesio a la madre si se presenta cualquiera de los siguientes: ● Presión arterial sistólica ≥140 o presión arterial diastólica ≥90, acompañado de cualquiera de los siguientes: Cefalea severa, alteraciones visuales como visión borrosa o fosfenos, dolor en hipocondrio derecho o epigástrico, vómito, hematuria, oliguria, trombocitopenia o sospecha de Síndrome Hellp. ● Presión arterial sistólica ≥160 o presión arterial diastólica ≥110 ● Eclampsia (Convulsiones) ● Edad gestacional ≤32 semanas con feto viable y trabajo de parto fase activa (neuroprotección fetal).
¿Antihipertensivo? ❏ No ❏ Sí, suministrado	Administre antihipertensivo según protocolo institucional si: ● La presión arterial sistólica es ≥160 o presión arterial diastólica es ≥ 110
¿Tratamiento para Sífilis? ❏ No, prueba negativa confirmada ❏ Sí, suministrado	Aplicar protocolo institucional. Si se desconoce el estado seguir protocolo institucional para realización de la prueba para sífilis y de acuerdo al resultado definir tratamiento
¿Antorretrovirales? ❏ No, VIH negativo confirmado ❏ Sí, suministrados	En caso de prueba de VIH positiva, administre antirretrovirales según protocolo institucional. Si se desconoce el estado, ordenar prueba rápida de VIH y de acuerdo al resultado definir manejo según protocolo institucional.
Suministros: ❏ Confirme la disponibilidad de suministros y el cumplimiento del protocolo para el lavado de manos. ❏ Confirme la disponibilidad y uso de guantes para cada examen vaginal.	Utilice guantes estériles en caso de membranas rotas
❏ Se alentó y se permite la presencia de un acompañante durante el trabajo parto. ❏ La gestante o acompañante reconocen los signos de alarma y solicitarán ayuda, en caso de: Sangrado, palidez, sudoración; dolor abdominal permanente, dolor de cabeza severo, problemas visuales o auditivos, problemas para orinar, sensación de pujo	
**Diligenciado por: __________________________________________________**	
Esta lista no pretende ser exhaustiva y no debe sustituir la historia clínica del paciente o el partograma. Se insta a realizar las modificaciones necesarias de la lista de verificación para que se adapte a las prácticas locales. Para obtener más información sobre el uso recomendado de la lista, por favor consulte la sección **“Safe Childbirth Checklist Manual”**, en: www.who.int/patientsafety	
**2. Justo antes del expulsivo (o antes de la cesárea)**	
**¿Se diligenció el partograma?** ❏ No ❏ Sí	Inicie el trazo cuando la dilatación cervical sea ≥4cm, ● Cada 30 minutos: registre la frecuencia cardiaca, contracciones y frecuencia cardiaca fetal. ● Cada 2 horas: registre la temperatura ● Cada 4 horas: registre la presión arterial
**La madre requiere:**	
¿Antibióticos? ❏ No ❏ Sí, suministrados	Suministre antibióticos a la madre si se presenta cualquiera de las siguientes: ● Temperatura ≥38ºC ● Flujo vaginal fétido ● Portadora desconocida o confirmada de Estreptococo Agalactiae ● Sospecha clínica de infección de cualquier localización ● Cesárea
¿Sulfato de Magnesio? ❏ No ❏ Sí, suministrado	Suministre sulfato de magnesio a la madre si se presenta cualquiera de los siguientes: ● Presión arterial sistólica ≥140 o presión arterial diastólica ≥90, acompañado de cualquiera de los siguientes: Cefalea severa, alteraciones visuales como visión borrosa o fosfenos, dolor en hipocondrio derecho o epigástrico, vómito, hematuria, oliguria, trombocitopenia o sospecha de Síndrome Hellp. ● Presión arterial sistólica ≥160 o presión arterial diastólica ≥110 ● Eclampsia (Convulsiones) ● Edad gestacional ≤32 semanas con feto viable y trabajo de parto fase activa (neuroprotección fetal).
¿Antihipertensivo? ❏ No ❏ Sí, suministrado	Administre antihipertensivo según protocolo institucional si: ● La presión arterial sistólica es ≥160 o presión arterial diastólica es ≥ 110
**Confirme si los suministros esenciales se encuentran disponibles para la atención del parto:**	
Para la madre ❏ Guantes estériles ❏ Antisépticos para el lavado de manos y preparación de la paciente según protocolo institucional ❏ Uterotónicos, de preferencia Oxitocina	Cuidados para la madre inmediatamente después del parto: ● Confirme el nacimiento de un solo bebé y no de un parto múltiple. ● Realice manejo activo del tercer estadío de acuerdo a protocolo institucional. ● Confirme que el útero está contraído.
Para el recién nacido ❏ Toalla limpia ❏ Bisturí o tijera estéril para cortar el cordón ❏ Ligadura de caucho, pinza plástica o cintas umbilicales estériles ❏ Fuente de calor ❏ Dispositivo de succión ❏ Bolsa-válvula-mascarilla	Cuidados inmediatos para el recién nacido: ● Secar al recién nacido y mantenerlo caliente. ● Si no respira: estimular y limpiar las vías respiratorias. ● Si después de eso continua sin respirar: - Sujetar y cortar el cordón - Ventilar con bolsa-válvula-mascarilla - Solicitar ayuda - Seguir protocolo institucional de reanimación neonatal
❏ En caso de ser necesario se dispone de un asistente (personal de salud/acompañante) para ayudar en la atención del parto	
Diligenciado por: __________________________________________________	
Esta lista no pretende ser exhaustiva y no debe sustituir la historia clínica del paciente o el partograma. Se insta a realizar las modificaciones necesarias de la lista de verificación para que se adapte a las prácticas locales. Para obtener más información sobre el uso recomendado de la lista, por favor consulte la sección **“Safe Childbirth Checklist Manual”**, en: www.who.int/patientsafety	

**Después del parto:** / LISTA DE VERIFICACIÓN DEL PARTO SEGURO

3. Después del parto (dentro de la primera hora)	
¿El sangrado de la madre es anormal?	Si el sangrado es anormal:
❏ No	● Masajee el útero
❏ Sí, active protocolo de manejo de hemorragia obstétrica institucional	● Utilice más uterotónicos
	● Inicie reanimación con líquidos endovenosos y considere hemoderivados
	● Trate la causa: tono, trauma, tejido, trombina (Nemotecnia cuatro T’s), atonía uterina, retención de restos placentarios, desgarro vaginal, ruptura uterina, coagulopatía
**La madre requiere:**	
¿Antibióticos?	Suministre antibióticos a la madre si se presenta cualquiera de las siguientes:
❏ No	● Parto instrumentado
❏ Sí, suministrados	● Extracción manual de la placenta.
	● Revisión uterina
	● Desgarro perineal severo
	● Temperatura ≥38ºC
	● Flujo vaginal fétido
	● Sospecha clínica de infección de cualquier localización
¿Sulfato de Magnesio?	Suministre sulfato de magnesio a la madre si se presenta cualquiera de los siguientes:
❏ No	● Presión arterial sistólica ≥140 o presión arterial diastólica mayor o igual a 90, acompañado de cualquiera de los siguientes: Cefalea severa, alteraciones visuales como visión borrosa o fosfenos, dolor en hipocondrio derecho o epigástrico, vómito, hematuria, oliguria, trombocitopenia o sospecha de Síndrome Hellp.
❏ Sí, suministrados	● Presión arterial sistólica ≥160 o presión arterial diastólica ≥110
	● Eclampsia (Convulsiones)
¿Antihipertensivo?	
❏ No	Administre antihipertensivo según protocolo institucional si:
❏ Sí, suministrado	● La presión arterial sistólica es ≥160 o presión arterial diastólica es ≥ 110
**El recién nacido requiere:**	
¿Ser remitido?	
❏ No	
❏ Sí, remitido	
¿Antibióticos?	Revise los criterios de remisión de su institución de salud
❏ No	Suministrar antibióticos al recién nacido si se le ha dado a la gestante por infección obstétrica o si presenta:}
❏ Si, suministrados	● Frecuencia respiratoria >60/min o <30/min
	● Retracción torácica, estertores o convulsiones
	● Pobre respuesta al estimulo
	● Temperatura <35ºC (sin incremento después de brindarle calor), o temperatura ≥38ºC
	● Sospecha de sífilis congénita
¿Cuidado especial o monitoreo?	Brindar cuidados especiales y monitoreo si el recién nacido presenta cualquiera de los siguientes:
❏ No	● Edad gestacional <37 semanas
❏ Sí, se ha organizado	● Peso al nacer <2500 gramos
	● Requirió antibióticos
	● Requirió reanimación neonatal
¿Antirretrovirales?	En caso de prueba de VIH positiva administre antirretrovirales según protocolo institucional para el recién nacido (la profilaxis debe comenzar dentro de las 12 horas después del parto)
❏ No	
❏ Sí, se ha organizado	
Se realizó tamizaje para hipotiroidismo congénito y hemoclasificación?	Tome la muestra de sangre neonatal según protocolo institucional
❏No, tome muestra	
❏Sí	
❏**Se inició la lactancia y el contacto piel a piel** (Si tanto la madre como el recién nacido se encuentran en buen estado de salud)	
❏**La gestante o acompañante reconocen los signos de alarma y solicitarán ayuda, en caso de:**	
Para la madre: Sangrado, palidez, sudoración; dolor abdominal permanente, dolor de cabeza severo, problemas visuales o auditivos, problemas	
para orinar, sensación de pujo.	
Para el recién nacido: dificultad para respirar/respiración rápida, fiebre, piel fría, succión débil, somnolencia o difícil de despertar.	
**Diligenciado por: ____________________________________________________**	
Esta lista no pretende ser exhaustiva y no debe sustituir la historia clínica del paciente o el partograma. Se insta a realizar las modificaciones necesarias de la lista de verificación para que se adapte a las prácticas locales. Para obtener más información sobre el uso recomendado de la lista, por favor consulte la sección **“Safe Childbirth Checklist Manual”**, en: www.who.int/patientsafety	
**4. Antes de dar el alta**	
**¿El sangrado de la madre está controlado?**	Si el sangrado es anormal:
❏No: tratar y posponer el alta	● Masajee el útero
❏Sí	● Utilice más uterotónicos
	● Inicie reanimación con líquidos endovenosos y considere hemoderivados
	● Trate la causa: tono, trauma, tejido, trombina (Nemotecnia cuatro T’s), atonía uterina, retención de restos placentarios, desgarro vaginal, ruptura uterina, coagulopatía
**¿La madre requiere antibióticos?**	Suministre antibióticos a la madre si se presenta cualquiera de las siguientes:
❏No	● Temperatura ≥38ºC en las últimas 24 horas
❏Sí: suministrar y posponer el alta	● Sangrado genital fétido
	● Sospecha clínica de infección de cualquier localización
	● Signos de infección del sitio quirúrgico superficial o profundo (episiorrafia, herida quirúrgica, endiometritis, miometritis, etc.)
**¿El recién nacido requiere antibióticos?**	Suministrar antibióticos al recién nacido si se presenta:
❏No	● Frecuencia respiratoria >60/min o <30/min
❏Sí: suministre antibióticos, retrase el alta, brinde cuidado especial y monitoreo.	● Retracción torácica, estertores o convulsiones
	● Pobre respuesta al estímulo
	● Temperatura <35ºC (sin incremento después de brindarle calor), o temperatura ≥38ºC
	● Dejó de lactar bien
	● Enrojecimiento periumbilical o drena pus
**¿El recién nacido está lactando bien?**	
	
❏No: retrase el alta hasta que establezca buenas prácticas de lactancia materna	
❏Sí	
**En caso de que la madre sea VIH positivo, ¿tanto la madre como el recién nacido tienen antirretrovirales (ARV) para 6 semanas?**	Si la madre es VIH positivo:
❏Sí	● Asigne cita de control por infectología o programa de VIH en consulta externa a la madre y al recién nacido antes de un mes
❏No, suspender lactancia materna y aplicar protocolo institucional	
❏**La madre y recién nacido recibieron tratamiento según el resultado de la serología**	
❏**Se retiraron catéteres y sondas, si fueron empleados**	
❏**Se ofreció consejería de planificación familiar y se refirió para la administración del método seleccionado**	
❏**Se asignó una cita de control**	
❏**La madre o acompañante reconocen los signos de alarma y solicitarán ayuda, en caso de:**	
**Signos de alarma en**	
**La madre:**	**El recién nacido:**
● Sangrado	● Dificultad para respirar / respiración rápida
● Dolor abdominal severo	● Fiebre
● Dolor de cabeza fuerte	● Piel fría
● Alteración visual	● Succión débil
● Dificultad para respirar	● Somnoliento o difícil de despertar
● Fiebre o escalofríos	● Tinte amarillo en ojos, piel, palmas de las manos o plantas de los pies
● Dificultad para orinar	
**Diligenciado por____________________________________________________**	
Esta lista no pretende ser exhaustiva y no debe sustituir la historia clínica del paciente o el partograma. Se insta a realizar las modificaciones necesarias de la lista de verificación para que se adapte a las prácticas locales. Para obtener más información sobre el uso recomendado de la lista, por favor consulte la sección **“Safe Childbirth Checklist Manual”**, en: www.who.int/patientsafety.
