# Supplementary material for: Adaptation and validation for Colombia of the WHO safe childbirth checklist
Source: Colomb Med (Cali). 2018 Sep 30;49(3):201–12. doi: 10.25100/cm.v49i2.2710 (PMC6220487; doi:10.25100/cm.v49i2.2710)
Supplement: Related texts

# Research article: Adaptación y validación de la lista de verificación del parto seguro de la OMS, para su uso en Colombia

## Introducción

La reducción de la mortalidad materna es una prioridad en salud pública a nivel mundial, definida como una de las “metas para el desarrollo del milenio”, de las Naciones Unidas ^1^. Según estadísticas de la Organización Mundial de la Salud (OMS) cada año se presentan aproximadamente 350,000 muertes maternas ^2^^,^^3^; la mayor parte de estas muertes ocurren en los países subdesarrollados o en vías de desarrollo y se considera que la mayoría de estos eventos son injustos y pueden ser evitados a través de intervenciones oportunas basadas en evidencia ^4^^,^^5^. Debido a esto, en la Agenda de Desarrollo Sostenible se pactó como Objetivo número 3.1 la meta de reducir la razón de mortalidad materna (RMM) mundial a menos de 70 por cada cien mil nacidos vivos, entre los años 2016 a 2030 ^6^.

En Colombia, para el año 2014, el Ministerio de Salud y de la Protección Social estimó con cifras del DANE una RMM de 53.7 por 100,000 nacidos vivos, sin embargo, los indicadores por departamento muestran grandes inequidades en el país, siendo los departamentos con mayores índices de pobreza los que tienen a su vez las RMM materna más altas, superando incluso por tres o cuatro veces al indicador nacional ^7^. Frente a este panorama se hace necesario redoblar esfuerzos para lograr los compromisos adquiridos, especialmente en las regiones con mayores inequidades en salud del país.

En años recientes se han adoptado con mayor frecuencia intervenciones basadas en listas de verificación, con el fin de ayudar en la gestión de tareas complejas o desatendidas en las que se corre el riesgo de cometer mayor número de errores. Estas consisten en una lista de elementos para ser comprobados durante una actividad compleja, como una ayuda de memoria, para asegurar la correcta ejecución de una tarea. La integración de los programas de las listas de verificación en la práctica clínica ha demostrado reducir la mortalidad y la incidencia de complicaciones en cirugía y cuidados intensivos ^8^^-^^11^.

Una estrategia basada en la lista de verificación se adapta bien a la atención del parto dadas varias características propias del evento, a saber: que las principales causas de mortalidad materna y perinatal están bien descritas; que la mayoría de muertes se producen dentro de una estrecha ventana de tiempo (veinticuatro horas después del nacimiento); que existen directrices internacionales para las mejores prácticas pero no se siguen; y algunas intervenciones probadas son relativamente económicas, costo-eficientes, fáciles de realizar, pero pueden ser difíciles de recordar y ejecutar en la secuencia apropiada, aspecto que podría solucionarse usan una lista de verificación ^12^^,^^13^.

Por esto, en el año 2008 la Organización Mundial de la Salud (OMS) estableció un programa de seguridad basado en la Lista de Verificación para Atención del Parto Seguro, con el objetivo de determinar si una intervención simple, de bajo costo, tenía impacto sobre la mortalidad materna y neonatal en los países de bajos ingresos. La lista (*The WHO Safe Childbirth Checklist),* inicialmente propuesta, contiene 29 ítems que abordan las principales causas mundiales de muerte materna (hemorragia, infección y trastornos hipertensivos); muerte fetal relacionada con el parto (atención intraparto inadecuada); y muerte neonatal (asfixia al nacer, infección y complicaciones relacionadas con la prematurez). Además, aborda la atención del nacimiento (por las dos vías: cesárea y parto vaginal), y controla simultáneamente todas las causas prevenibles, directas e indirectas de mortalidad materna, en las primeras 24 horas desde el nacimiento hasta el egreso hospitalario.

Los resultados de las pruebas piloto mostraron que la implementación de la lista de verificación del parto llevó a mejorar la calidad de la atención prestada por los trabajadores de la salud que atienden los partos institucionales ^14^, además de ser económica, fácil de realizar y con evidencia de buenos resultados en 10 países de África y Asia ^5^.

Debido a estos resultados, en el año 2012 la OMS emprendió una iniciativa que implicó la colaboración de 29 países y 34 grupos de investigación, iniciativa que principalmente buscaba evaluar los factores que facilitaban o dificultaban la aplicación y uso efectivo de esta herramienta y que llevó a diseñar un guía de implementación y una actualización de la lista de verificación ^15^. En Colombia no se ha implementado aún y son necesarios estudios que determinen su aplicabilidad y aceptación en nuestro medio.

Sin embargo, para incorporar este instrumento a una población diferente, su formato debe modificarse para que se adapte al medio en que será aplicado. Por ende, en el marco del estudio piloto a nivel mundial, en nuestro país se debió hacer la traducción y adaptación de la lista a nuestro medio. El presente estudio pretendió traducir, adaptar y validar el contenido de la Lista de Verificación del Parto Seguro de la OMS para su uso en Colombia

## Materiales y Métodos

### Diseño

Estudio de validación de instrumentos de medición.

### Selección y descripción de los participantes

Para el consenso de expertos se contó con un equipo de 17 expertos en seguridad del paciente, obstetricia, en seguridad y mortalidad materna y neonatal: 6 anestesiólogos, 1 internista, 5 ginecobstetras, 4 enfermeras y 1 pediatra neonatólogo. La mayoría de estos tenían más de 10 años de experiencia, sólo 2 tenían menos de ese tiempo de experiencia (7 y 2 años), fueron seleccionados por referenciación de expertos. El listado de jueces expertos se puede ver en el Anexo 1.

### Procedimiento

En el proceso de adaptación del instrumento se siguieron las indicaciones hechas por la Asociación de Psicólogos Americana (A.P.A.) para la adaptación de instrumentos de medición, que incluyen: 1) asegurarse de que existe una equivalencia entre los idiomas y grupos culturales de interés, 2) decidir si se adapta un instrumento ya existente o se desarrolla uno nuevo, 3) seleccionar traductores profesionales cualificados, 4) realizar traducción directa o inversa, 5) revisar la versión adaptada del instrumento y realizar las correcciones necesarias, entre otros ^16^^,^^17^. Teniendo en cuenta estas recomendaciones, en el presente estudio se siguieron las siguientes fases:

#### Fase 1. Traducción

Se realizó la traducción del instrumento original con ayuda de un traductor cualificado nativo de Colombia, esta versión se revisó entre los co-investigadores del proyecto. De acuerdo con las sugerencias y observaciones realizadas en esta fase se hicieron los últimos ajustes a la traducción del mismo.

#### Fase 2. Validez de contenido

Esta generalmente es evaluada a través de un panel o juicio de expertos, el cual está definido como “una opinión informada de personas con trayectoria en el tema, que son reconocidas por otros como expertos cualificados en éste, y que pueden dar información, evidencia, juicios y valoraciones” ^18^. En este estudio, la validez de contenido se analizó por medio de consenso de evaluadores aplicando la metodología de discusión entre los expertos y de consenso a partir de un Delphi modificad ^19^.

Al grupo de jueces se les entregó un formato para evaluar la claridad y relevancia de cada uno de los ítems que componían la lista de verificación y la suficiencia de estos para medir cada fase de la atención del parto propuestos en el instrumento original. Las puntuaciones dadas a cada comportamiento o apartado, en cada una de estas categorías, oscilaron en una escala Likert de 1 a 4, de acuerdo a los criterios que se muestran en la Tabla 1.

**Tabla 1:** Definición de cada categoría y puntajes a aplicar en la valoración de los ítems y apartados de la lista.

Claridad (Cla)
El ítem se comprende fácilmente, es decir, su sintáctica y semántica son adecuadas
1. El ítem no es claro
2. El ítem requiere bastantes modificaciones o una modificación muy grande en el uso de las palabras de acuerdo con su significado o por la ordenación de las mismas.
3. Se requiere una modificación muy específica de algunos de los términos del ítem.
4. El ítem es claro, tiene semántica y sintaxis adecuada
**Relevancia (Rel)**
El ítem que está revisando es importante para el momento en que se está aplicando la lista de verificación, en este caso corresponde con un aspecto importante y necesario de verificar en este punto del trabajo de parto, y por tanto debe ser incluido en el instrumento.
1. El ítem puede ser eliminado
2. El ítem tiene alguna relevancia, pero otro ítem puede incluir lo que mide éste
3. El ítem es importante pero no es determinante para ser incluido.
4. El ítem es muy importante, debe ser incluido.
**Suficiencia (Suf)**
Los ítems que pertenecen a un mismo momento en la lista de verificación bastan y son suficientes para cubrir los aspectos esenciales a verificar en el trabajo de parto.
1. Los ítems no son suficientes
2. Los ítems cubren algo, pero no en su totalidad, se deben añadir o cambiar ítems.
3. Se deben añadir o cambiar pocos ítems para poder cubrir los aspectos esenciales a verificar en el trabajo de parto
4. Los ítems son suficientes para obtener una medición completa de los aspectos esenciales a verificar en el trabajo de parto

Se calculó la fiabilidad entre jueces (𝒓_𝑾𝑮_ *: within-group interrater reliability*) para cada comportamiento; la cual se usa para determinar el nivel de acuerdo entre evaluadores en la validación de ítems, y se calcula a partir de la fórmula:

$$r_{WG}\;=\;1\;-\left(\frac{S_{Xj}^{2}}{\sigma_{EU}^{2}}\right)$$

Donde 𝒓_𝑾𝑮_ indica la fiabilidad entre evaluadores, en un grupo de *k* evaluadores para un ítem

$X_{j}:\;S_{Xj}^{2}$ es la varianza observada de $X_{j}\;y\;\sigma_{EU}^{2}$ es la varianza esperada por azar de X_j_, es decir en una condición de fiabilidad entre evaluadores igual cero ^20^.

De acuerdo con este cálculo, se decidió eliminar, modificar o no modificar cada ítem y añadir o no nuevos ítems a cada fase de la lista. El criterio que se acepta es que si el nivel de acuerdo (R_wg_) no supera un punto de corte de 0.8, se deben modificaciones al ítem o al instrumento ^21^. En este caso un R_wg_ menor a 0.8 en claridad indicaba la necesidad de modificar el ítem, un R_wg_ menor a 0.8 en relevancia abría la discusión sobre eliminar o no el ítem y un R_wg_ menor a 0.8 en suficiencia abría la discusión sobre los ítems que era necesario incluir en la versión para Colombia. Este proceso se realizó varias veces hasta que las modificaciones realizadas mostraron un acuerdo entre los jueces superior al punto de corte.

### Instrumento

La lista de Verificación del Parto Seguro (LVPS) fue desarrollada por la OMS en un trabajo colaborativo con enfermeras, parteras, obstetras, pediatras, expertos en seguridad del paciente y pacientes de todo el mundo; como una estrategia para ayudar a los trabajadores de la salud a mejorar sus prácticas en la atención materna y del recién nacido. Se enfoca en las principales causas de mortalidad materna en el mundo (hemorragia, infección, trastorno hipertensivo y trabajo de parto disfuncional), mortinatos relacionados con el intraparto (atención inadecuada) y muertes neonatales (eventos durante el trabajo de parto, infecciones y complicaciones de prematuridad). Cada ítem de la lista es una acción crítica y su omisión puede conducir a situaciones graves.

Las pruebas piloto han mostrado que la LVPS (versión 1.0), ha mejorado las prácticas de los trabajadores de la salud ^5^. Está propuesta para ser utilizada en cuatro puntos de tiempo (puntos de pausa). En cada punto se debe completar un conjunto de prácticas esenciales (listado de ítems). Debe utilizarse una lista para cada madre y su recién nacido, en la que cada ítem es comprobado marcándolo cuando éste se ha completado o realizado. Los responsables de llenarla pueden ser enfermeras, parteras, médicos u otros trabajadores de la salud.

Los puntos de pausa suceden en aquellas situaciones críticas en las que se pueden evitar complicaciones o tratarse adecuadamente. También tienen lugar en aquellos momentos en los que es conveniente revisar a la madre y al recién nacido. De este modo, la Lista de Verificación del Parto Seguro está diseñada para utilizarse en estos cuatro puntos de pausa durante los partos institucionales:

1. En el ingreso (8 ítems)
2. Justo antes del trabajo de parto o antes de la cesárea (5 ítems)
3. Después del nacimiento, dentro de la primera hora (9 ítems)
4. Y, antes de dar de alta (7 ítems)

### Consideraciones éticas

La realización del presente trabajo se adecua a las recomendaciones para investigación biomédica nacionales e internacionales ^22^^,^^23^. El protocolo de investigación fue aprobado por el Comité de Ética de la Universidad Nacional de Colombia.

### Control de sesgos

Para controlar posibles sesgos debidos a las influencias entre los miembros del consenso se decidió aplicar un Delphi modificado, en el cual en primera instancia los expertos dieron las puntuaciones de forma individual y cegada a los puntajes de los otros expertos, y en segunda instancia en la jornada de consenso presencial se usaron dispositivos de puntuación que fueron entregados a cada experto de modo que cada uno volvía a dar los puntajes que considerara de forma individual y cegada a los puntajes de los otros expertos.

## Resultados

Una vez determinada la fiabilidad entre los jueces (r_WG_) para cada ítem y fase de atención, y teniendo en cuenta los criterios de decisión, se hicieron modificaciones a la redacción de 28 ítems, con el fin de hacerlos más acordes para el uso en nuestro contexto; no se eliminaron ítems, y se dejó 1 ítem tal como estaba, los resultados se presentan en la Figura 1 y la Tabla 2.

**Figura 1:** Diagrama de flujo de la validación de contenido de la lista de verificación del parto seguro para Colombia

**Tabla 2:** Porcentaje de acuerdo en las categorías “claridad y coherencia” y decisión final frente a cada ítem

No	Ítem inicial		Claridad rWG	Relevancia rWG	Decisión	Ítem final
Antes del Parto							
1	¿La madre necesita ser remitida?	Verifique los criterios de remisión de su institución	0.71	1.00	Modificar	¿La madre necesita ser remitida?	Verifique los criterios de remisión de su institución
	❏ No					❏ No	
	❏Sí, se ha organizado				No Eliminar	❏ Sí, se iniciaron trámites correspondientes	
2	¿Se inició el partograma?	Comience a trazar cuando el cérvix sea ≥ 4cm,	0.62	1.00	Modificar	¿Se inició el partograma?	Inicie el trazo cuando la dilatación cervical sea ≥4cm,
	❏ No: iniciará a los ≥4cm	luego el cérvix debe dilatarse ≥ 1 cm / hora				❏ No: Se iniciará cuando la dilatación cervical sea ≥4cm	● Cada 30 minutos: registre la frecuencia cardiaca y frecuencia cardiaca fetal (FCF). En el expulsivo la FCF se debe tomar cada 10 minutos
	❏ Sí	● Cada 30 min.: Trace el FC, contracciones, FCF				❏ Sí	● Cada hora registre: signos vitales y actividad uterina
		● Cada 2 horas: Trace la temperatura			No Eliminar		● Cada 2 horas: registre la presión arterial
		● Cada 4 horas: Trace la presión arterial					● Cada 4 horas: registre la temperatura
3	¿Antibióticos?	Suministrar antibióticos a la madre si se presenta:	0.45	0.86	Modificar	¿Antibióticos?	Suministre antibióticos a la madre si se presenta cualquiera de las siguientes:
	❏ No	● Temperatura ≥38°C				❏ No	● Temperatura ≥38ºC
	❏ Sí, suministrados	● Flujo vaginal con mal olor				❏ Sí, suministrados	● Flujo vaginal fétido
		● Ruptura de las membranas >18 hrs			No Eliminar		● Portadora confirmada de Estreptococo Agalactiae
							● Portadora desconocida de Estreptococo Agalactiae con edad gestacional menor a 37 semanas o ruptura membranas mayor a 18 horas.
							● Sospecha clínica de infección de cualquier localización
4	¿Sulfato de Magnesio?	Suministrar Sulfato de Magnesio a la madre si se presenta:	0.44	0.88	Modificar	¿Sulfato de Magnesio?	Suministre sulfato de magnesio a la madre si se presenta cualquiera de los siguientes:
	❏ No	● Presión diastólica BP ≥ 110mmHg y 3+ de proteinuria				❏ No	● Presión arterial sistólica ≥140 o presión arterial diastólica ≥90, acompañado de cualquiera de los siguientes: Cefalea severa, alteraciones visuales como visión borrosa o fosfenos, dolor en hipocondrio derecho o epigástrico, vómito, hematuria, oliguria, trombocitopenia o sospecha de Síndrome Hellp.
	❏ Sí, suministrado	● Presión diastólica BP ≥ 90 MMhG, 2+ de proteinuria y algo de lo siguiente: fuerte dolor de cabeza, alteración visual, dolor epigástrico.				❏ Sí, suministrado	● Presión arterial sistólica ≥160 o presión arterial diastólica ≥110
					No Eliminar		● Eclampsia (Convulsiones)
							● Edad gestacional ≤32 semanas con feto viable y trabajo de parto fase activa (neuroprotección fetal).
5	¿Antorretrovirales?	Las madres con CD4 ≤350 o clínicamente diagnosticadas con el virus requieren tratamiento Las madres con CD4 >350 requieren profilaxis	0.52	0.92	Modificar	¿Antirretrovirales?	En caso de prueba de VIH positiva, administre antirretrovirales según protocolo institucional. Si se desconoce el estado, ordenar prueba rápida de VIH y de acuerdo al resultado definir manejo según protocolo institucional.
	❏ No, se confirma VIH negativo					❏ No, VIH negativo confirmado	
	❏ Sí, suministrados				No Eliminar	❏ Sí, suministrados	
	Si se desconoce el estado, ordenar prueba de VIH						
6	Confirme la disponibilidad de suministros para la asepsia de las manos y uso de guantes para cada examen vaginal.		0.62	1.00	Modificar	Confirme la disponibilidad de suministros y el cumplimiento del protocolo para el lavado de manos.	Utilice guantes estériles en caso de membranas rotas
					No Eliminar	Confirme la disponibilidad y uso de guantes para cada examen vaginal	
7	Motive la presencia de un acompañante en el momento del parto.		0.48	0.88	Modificar	>Se alentó y se permite la presencia de un acompañante durante el trabajo parto	
					No Eliminar		
8	Confirme que la madre/acompañante solicitarán ayuda durante el trabajo de parto, en caso de:	● Sangrado	0.32	0.96	Modifica	La gestante o acompañante reconocen los signos de alarma y solicitarán ayuda, en caso de: Sangrado, palidez, sudoración; dolor abdominal permanente, dolor de cabeza severo, problemas visuales o auditivos, problemas para orinar, sensación de pujo	
		● Dolor abdominal severo					
		● Dolor de cabeza severo o problemas visuales			No Eliminar		
		● problemas para orinar					
		● Necesidad de pujar					
**Justo antes del parto (o antes de la cesárea)**							
9	La madre requiere:	Suministrar antibióticos a la madre si se presenta:	0.36	0.92	Modificar No Eliminar	La madre requiere:	Suministre antibióticos a la madre si se presenta cualquiera de las siguientes:
	¿Antibióticos?	● Temperatura ≥38°C				¿Antibióticos?	● Temperatura ≥38ºC
	❏ No	● Flujo vaginal con mal olor				❏ No	● Flujo vaginal fétido
	❏ Sí, suministrados	● Ruptura de las membranas >18 hrs				❏ Sí, suministrados	● Portadora desconocida o confirmada de Estreptococo Agalactiae
		● Cesárea					● Sospecha clínica de infección de cualquier localización
							● Cesárea
							● Parto en condiciones no seguras
10	¿Sulfato de Magnesio?	Suministrar Sulfato de Magnesio a la madre si se presenta:	0.38	0.92	Modificar	¿Sulfato de Magnesio?	Suministre sulfato de magnesio a la madre si se presenta cualquiera de los siguientes:
	❏ No	● Presión diastólica BP ≥ 110mmHg y 3+ de proteinuria				❏ No	● Presión arterial sistólica ≥140 o presión arterial diastólica ≥90, acompañado de cualquiera de los siguientes: Cefalea severa, alteraciones visuales como visión borrosa o fosfenos, dolor en hipocondrio derecho o epigástrico, vómito, hematuria, oliguria, trombocitopenia o sospecha de Síndrome Hellp.
	❏ Sí, suministrado	● Presión diastólica BP ≥ 90 MMhG, 2+ de proteinuria y si presenta fuerte dolor de cabeza, dolor epigástrico, o alteraciones visuales.				❏ Sí, suministrado	● Presión arterial sistólica ≥160 o presión arterial diastólica ≥110
					No Eliminar		● Eclampsia (Convulsiones)
							● En pacientes con pre eclampsia severa continúe el sulfato de magnesio hasta por 24 horas después del parto.
11	Para la madre	Cuidados para la madre inmediatamente después del parto:	0.27	0.96	Modificar	Para la madre	Cuidados para la madre inmediatamente después del parto:
	Guantes	Confirmar el nacimiento de un solo bebé y no de un parto múltiple.				Guantes estériles	● Confirme el nacimiento de un solo bebé y no de un parto múltiple.
	Desinfecta nte a base de alcohol, jabón y agua limpia	1. Suministro de oxitocina 1 minuto después del nacimiento.				Antisépticos para el lavado de manos y preparación de la paciente según protocolo institucional	● Realice manejo activo del tercer estadío de acuerdo a protocolo institucional.
	10 unidades de oxitocina en una jeringa	2. Extracción de la placenta			No Eliminar	Uterotónicos, de preferencia Oxitocina	● Confirme que el útero está contraído.
		3. Confirmación de que el útero está contraído.					
12	Para el bebé	Cuidados inmediatos para el recién nacido:	0.41	1.00	Modificar	Para el recién nacido	Cuidados inmediatos para el recién nacido:
	Toalla limpia	1. Secar al bebé y mantenerlo caliente				Toalla limpia	● Secar al recién nacido y mantenerlo caliente.
	Bisturí estéril para cortar el cordón	2. Si no respira: estimular y limpiar las vías respiratorias				Bisturí o tijera estéril para cortar el cordón	● Si no respira: estimular y limpiar las vías respiratorias.
	Dispositivo de succión	3. Si después de eso continua sin respirar:				Ligadura de caucho, pinza plástica o cintas umbilicales estériles	● Si después de eso continua sin respirar:
	Bolsa-válvula- mascarilla	-sujetar y cortar el cordón			No Eliminar	Fuente de calor	- Sujetar y cortar el cordón
		-ventilar con bolsa- válvula-mascarilla				Dispositivo de succión	- Ventilar con bolsa-válvula- mascarilla
		-Solicite ayuda				Bolsa-válvula-mascarilla	- Solicitar ayuda
							- Seguir protocolo institucional de reanimación neonatal
13	¿En caso de ser necesario, se encuentra el asistente listo para ayudar en la labor de parto?		0.51	0.57	Modificar	En caso de ser necesario se dispone de un asistente (personal de salud/acompañante) para ayudar en la atención del parto	
					Luego de la deliberación se decidió No Eliminar		
**Después del parto (dentro de la primera hora)**							
14	¿El sangrado de la madre es anormal?	Si el sangrado es anormal:	0.45	1.00	Modificar	¿El sangrado de la madre es anormal?	Si el sangrado es anormal:
	❏ No	· Masajeen el útero				❏ No	● Realizar hemoclasificación
	❏ Sí, pedir ayuda	● Considere más uterotónicos				❏ Sí, active protocolo de manejo de hemorragia obstétrica institucional	● Masajee el útero
		● Inicie IV			No Eliminar			● Utilice más uterotónicos
		● Trate la causa: atonía uterina, retención de restos placentarios, desgarro vaginal, ruptura uterina					● Inicie reanimación con líquidos endovenosos y considere hemoderivados
							● Trate la causa: tono, trauma, tejido, trombina (Nemotecnia cuatro T’s), atonía uterina, retención de restos placentarios, desgarro vaginal, ruptura uterina, coagulopatía.
15	La madre requiere:	Suministrar antibióticos a la madre si la placenta se ha extraído manualmente o si la madre presenta una temperatura ≥38°C o alguno de los siguientes síntomas:	0.56	0.92	Modificar	La madre requiere:	Suministre antibióticos a la madre si se presenta cualquiera de las siguientes:
	¿Antibióticos?	● Escalofríos				¿Antibióticos?	● Parto instrumentado
	❏ No	● Flujo vaginal con mal olor				❏ No	● Extracción manual de la placenta.
	❏ Sí, suministrados					❏ Sí, suministrados	● Revisión uterina
					No Eliminar		● Desgarro perineal severo
							● Temperatura ≥ 38ºC
							● Flujo vaginal fétido
							● Sospecha clínica de infección de cualquier localización
16	¿Sulfato de Magnesio?	Suministre sulfato de magnesio a la madre si se presenta:	0.62	0.60	Modificar Luego de la deliberación se decidió No Eliminar	¿Sulfato de Magnesio?	Suministre sulfato de magnesio a la madre si se presenta cualquiera de los siguientes:
	❏ No	● Presión diastólica BP ≥ 110mmHg y 3+ de proteinuria				❏ No	● Presión arterial sistólica ≥140 o presión arterial diastólica mayor o igual a 90, acompañado de cualquiera de los siguientes: Cefalea severa, alteraciones visuales como visión borrosa o fosfenos, dolor en hipocondrio derecho o epigástrico, vómito, hematuria, oliguria, trombocitopenia o sospecha de Síndrome Hellp.
	❏ Sí, suministrado	● Presión diastólica BP ≥ 90 mmHg, 2+ de proteinuria y algo de lo siguiente: fuerte dolor de cabeza, alteración visual o dolor epigástrico.				❏ Sí, suministrados	● Presión arterial sistólica ≥160 o presión arterial diastólica ≥110
							● Eclampsia (Convulsiones)
							● Continuar magnesio por 24 horas en paciente con preclamsia severa
17	El bebé requiere:	Revise los criterios de remisión de su institución de salud	0.84	1.00	No Modificar	El bebé requiere:	Revise los criterios de remisión de su institución de salud
	¿Ser remitido?					¿Ser remitido?	
	❏ No				No Eliminar	❏ No	
	❏ Sí, remitido					❏ Sí, remitido	
18	¿Antibióticos?	Suministrar antibióticos al bebé,	0.62	0.96	Modificar	¿Antibióticos?	Suministrar antibióticos al recién nacido
	❏ No	si se le ha dado a la madre o si el bebé presenta:				❏ No	si se le ha dado a la gestante por infección obstétrica o si presenta:
	❏ Sí, suministrados	● Rango de respiración de >60/min o <30/min				❏ Sí, suministrados	● Frecuencia respiratoria >60/min
		● Retracción torácica, estertores o convulsiones					● Retracción torácica o estertores
		● Pobre movimiento al estimulo					● Pobre respuesta al estimulo
		● Temperatura < 35° C (sin incremento después de brindarle calor), o temperatura ≥ 38°			No Eliminar		● Temperatura <35º C (sin incremento después de brindarle calor), o temperatura ≥38º C
							● Sospecha de sífilis congénita
							● APGAR menor de 7
							Nota: NO se debe aplicar antibióticos en pacientes con FR < 30/min o convulsiones
19	¿Cuidado/monitoreo especial?	Brindar cuidados especiales y monitoreo si el bebé:	0.72	1.00	Modificar	¿Cuidado especial o monitoreo?	Brindar cuidados especiales y monitoreo si el recién nacido presenta cualquiera de los siguientes:
	❏ No	● Tiene más de un mes de prematuridad				❏ No	● Edad gestacional <37 semanas
	❏ Sí, se ha organizado	● Pesa <2,500 g al nacer			No Eliminar	❏ Sí, se ha organizado	● Peso al nacer <2,500 gramos
		● Requiere antibióticos					● Requirió antibióticos
		● Requiere reanimación neonatal					● Requirió reanimación neonatal
20	¿Antirretrovirales?	Si la madre es VIH+, seguir los lineamientos locales de la institución para el bebé (la profilaxis debe comenzar dentro de las 12 h después del parto)	0.71	0.96	Modificar	¿Antirretrovirales?	En caso de prueba de VIH positiva administre antirretrovirales según protocolo institucional para el recién nacido (la profilaxis debe comenzar dentro de las 12 horas después del parto)
	❏ No					❏ No	
	❏ Sí, se ha organizado				No Eliminar	❏ Sí, se ha aplicado	
21	Inició la lactancia y el contacto piel a piel (Siempre y cuando tanto la madre como el bebé se encuentren en buen estado de salud)		0.72	1.00	Modificar No Eliminar	Se inició la lactancia y el contacto piel a piel (Si tanto la madre como el recién nacido se encuentran en buen estado de salud)	
22	Confirmar que la madre o el acompañante solicitarán ayuda si se presentan señales de peligro		0.59	0.55	Modificar Luego de la deliberación se decidió No Eliminar	La gestante o acompañante reconocen los signos de alarma y solicitarán ayuda, en caso de:	
						Para la madre: Sangrado, palidez, sudoración; dolor abdominal permanente, dolor de cabeza severo, problemas visuales o auditivos, problemas para orinar, sensación de pujo.	
						Para el recién nacido: dificultad para respirar/respiración rápida, fiebre, piel fría, succión débil, somnolencia o difícil de despertar.	
**Antes del alta**							
23	¿El sangrado de la madre está controlado?		0.53	0.62	Modificar Luego de la deliberación se decidió No Eliminar	¿El sangrado de la madre está controlado?	Si el sangrado es anormal:
	❏ No: tratar y posponer el alta					❏ No: tratar y posponer el alta	● Masajee el útero
	❏ Sí					❏ Sí	● Utilice más uterotónicos
							● Inicie reanimación con líquidos endovenosos y considere hemoderivados
							● Trate la causa: tono, trauma, tejido, trombina (Nemotecnia cuatro T’s), atonía uterina, retención de restos placentarios, desgarro vaginal, ruptura uterina, coagulopatía
24	¿La madre requiere antibióticos?	Suministre antibióticos a la madre si su temperatura es ≥38°C o si presenta:	0.62	0.92	Modificar No Eliminar	¿La madre requiere antibióticos?	Suministre antibióticos a la madre si se presenta cualquiera de las siguientes:
	❏ No	· Escalofríos				❏ No	● Temperatura ≥38ºC en las últimas 24 horas
	❏ Sí: suministrar y posponer el alta	● Flujo vaginal con mal olor				❏ Sí: suministrar y posponer el alta	● Sangrado genital fétido
							● Sospecha clínica de infección de cualquier localización
							● Signos de infección del sitio quirúrgico superficial o profundo (episiorrafia, herida quirúrgica, endometritis, miometritis, etc.)
25	¿El bebé requiere antibióticos?	Suministre antibióticos al bebé si presenta:	0.71	1.00	Modificar No Eliminar	¿El recién nacido requiere antibióticos?	Suministrar antibióticos al recién nacido si se presenta:
	❏ No	● Frecuencia respiratoria >60/min o <30/min				❏ No	● Frecuencia respiratoria >60/min o <30/min
	❏ Sí: suministrar antibióticos, retrasar el alta, dar cuidado especial.	● Retracción torácica, estertores o convulsiones				❏ Sí: suministre antibióticos, retrase el alta, brinde cuidado especial y monitoreo.	● Retracción torácica, estertores o convulsiones
		● Pobre movimiento al estímulo					● Pobre respuesta al estímulo
		● Temperatura < 35°C (sin incremento después de brindarle calor) o temperatura ≥ 38°					● Temperatura <35ºC (sin incremento después de brindarle calor), o temperatura ≥38ºC
		● Dejó de lactar bien					● Dejó de lactar bien
		● El enrojecimiento umbilical se extiende a la piel o drena pus					● Enrojecimiento peri umbilical o drena pus
26	¿El bebé está lactando bien?		0.71	1.00	Modificar No Eliminar	¿El recién nacido está lactando bien?	
	❏ No: establecer buenas prácticas de lactancia materna hasta dar de alta					❏ No: retrase el alta hasta que establezca buenas prácticas de lactancia materna	
	❏ Sí					❏ Sí	
27	En caso de que la madre sea VIH positivo, ¿tanto la madre como el bebé tienen antirretrovirales (ARV) para 6 semanas?		0.62	0.92	Modificar No Eliminar	En caso de que la madre sea VIH positivo, ¿tanto la madre como el recién nacido tienen antirretrovirales (ARV) para 6 semanas?	Si la madre es VIH positivo:
	❏ Sí					❏ Sí	● Asigne cita de control por infectología o programa de VIH en consulta externa a la madre y al recién nacido antes de un mes
						❏ No, suspender lactancia materna y aplicar protocolo institucional	
28	Discutir y ofrecer opciones de planificación familiar a la madre		0.73	0.95	Modificar No Eliminar	Se ofreció asesoría de planificación familiar y se refirió para la administración del método seleccionado
29	Realizar seguimiento y confirmar que la madre o el acompañante solicitarán ayuda si se presentan señales de peligro después de dar de alta.		0.78		1.00	Modificar No Eliminar	La madre o acompañante reconocen los signos de alarma y solicitarán ayuda, en caso de:	
							Para la madre: Sangrado, dolor abdominal severo, dolor de cabeza fuerte, alteración visual, dificultad para respirar, fiebre o escalofríos, dificultad para orinar.	
							Para el recién nacido: dificultad para respirar/respiración rápida, fiebre, piel fría, succión débil, somnoliento o difícil de despertar, tinte amarillo en ojos, piel, palmas de las manos o plantas de los pies.	

Como puede observarse en la Tabla 2, la mayor parte de modificaciones importantes se hicieron a las orientaciones de manejo que acompañan cada ítem y algunas pequeñas modificaciones a la redacción o los términos usados en estos.

Además de esto, se agregaron 19 nuevos ítems: 4 en la fase de ingreso, 9 antes del parto, 2 justo después del parto y 4 en la fase de egreso. Los 4 ítems de la fase de ingreso se refieren a revisión de alergias, necesidad de antihipertensivo y tratamiento para sífilis y la división del ítem de suministros en dos partes. Los 9 ítems que se agregaron al apartado antes del parto se deben a que el ítem de suministros se contaba como uno sólo y en la versión nueva se dejaron como cada uno aparte, además se agregaron preguntas sobre el partograma y la necesidad de antihipertensivo. Justo después del parto se agregaron dos ítem sobre necesidad de antihipertensivo para la madre y tamizaje para hipotiroidismo congénito y hemoclasificación para el recién nacido. Finalmente, antes del alta se agregaron 4 ítems que se refieren a indagar si se evidencia síndrome anémico en la madre, si se asignó una cita de control postparto, si se dio tratamiento para sífilis y si se retiraron catéteres y sondas empleados.

La versión final de la Lista de Verificación del Parto Seguro para su uso en Colombia puede verse en el Anexo 2

## Discusión

La lista de chequeo para la atención del parto ^5^, como su homóloga en cirugía ^8^, es una herramienta para optimizar los procesos estandarizados del personal de la salud, garantizándole al clínico tener en cuenta eventos y acciones que frente a cada etapa del parto podrá requerir para hacer su atención a la paciente de mejor calidad. No constituye una guía de práctica clínica, aun así, otorga un estándar mínimo de cuidado, al favorecer en cada parto la valoración y toma de conductas básicas pertinentes a cada atención, aportando significativamente a la seguridad clínica de la paciente y a la seguridad jurídica del personal que presta la atención. Para incorporar al máximo estos beneficios, se requiere que la herramienta sea adaptada al contexto en donde será aplicada ^14^^,^^15^, por lo que se expuso en este artículo su adaptación y validación a nuestro medio.

Así, siguiendo la metodología descrita, tras la traducción del original y luego de revisar y ajustar la redacción en español de cada pregunta y sus recomendaciones, en consenso se modificaron y adicionaron puntos en cada uno de los cuatro momentos de la lista. En el primer momento, el del ingreso, se modificaron todos sus puntos, adicionando consideraciones a cada pregunta, y se formularon cuatro nuevas; así, acerca del inicio del partograma, se modificaron los tiempos sugeridos para la toma de signos. La propuesta de tomar la presión arterial cada dos horas en lugar de cada cuatro se consideró debido a que cada cuatro horas es un lapso muy largo de tiempo para valorar el impacto de posibles conductas que se pudieran tomar. En cuanto a la medición de la temperatura cada cuatro horas en lugar de cada dos, se debió a que se consideró que la fiebre durante el trabajo de parto es infrecuente y a que este parámetro no es tan variable como para buscar sus alteraciones tan frecuentemente. Se requiere también la toma de la frecuencia cardiaca materna y fetal cada 30 min y la medición de la actividad uterina cada hora, puesto que estos parámetros presentan rápida variación con el cambio de las condiciones clínicas y con las intervenciones en la paciente y neonato.

En cuanto al uso de antibióticos para la madre, se realizaron modificaciones en varios de los momentos de la lista, así, al momento del ingreso, se agregó el inicio de antibioticoterapia en las pacientes portadoras confirmadas de *S. Agalactiae*^24^ y en las que se tenga sospecha de una gestación menor de 37 semanas o con ruptura de membranas de 18 horas, debido a que esta infección conlleva con mucha frecuencia a una sepsis neonatal temprana, que en muchos de los casos conduce a la muerte neonatal. Por último, se aclara que, ante la sospecha clínica de una infección en la madre, en cualquier localización debe iniciarse la antibioticoterapia respectiva, puesto que dicha infección podría complicar el parto con resultados desfavorables para la madre y/o al neonato. Respecto al uso de sulfato de magnesio se ampliaron las consideraciones para su administración, especialmente su uso en caso de eclampsia ^25^.

Se adicionaron nuevas preguntas: si ¿La madre es alérgica? Desde el inicio de la atención del parto es fundamental conocer si la paciente, al recibir un medicamento, puede presentar una reacción alérgica que se manifieste como un choque anafiláctico e incluso causar la muerte. La segunda hace referencia a: si ¿La madre requiere tratamiento para sífilis? Considerando la alta prevalencia de esta enfermedad en nuestro medio y su posibilidad de transmisión al feto, protegiendo así a la madre y previniendo la sífilis congénita. El tratamiento para sífilis con antibioticoterapia antitreponémica es categórico y determinante si se diagnostica sífilis a la madre ^26^. Si se desconoce el estado con respecto a la sífilis, se debe seguir el protocolo institucional para la realización de la prueba para sífilis y de acuerdo al resultado definir el tratamiento. La tercera pregunta: si ¿La madre requiere manejo antihipertensivo? La hipertensión gestacional podría causar morbimortalidad materna y fetal; predisponiendo al desarrollo de complicaciones potencialmente mortales como desprendimiento de placenta, hemorragia cerebral, insuficiencia hepática, renal y coagulación intravascular diseminada ^27^.

En el segundo momento “justo antes del expulsivo o de la cesárea”, se adicionaron preguntas, una referente al inicio de antihipertensivos y otra referente a la evolución del partograma. Su diligenciamiento desde el ingreso, tiene como resolución la predicción de complicaciones y de la necesidad de intervenciones justo en el momento del nacimiento. Al apartado que se refiere a la antibioticoterapia para la madre, se adicionó la sospecha o infección por *S. Agalactiae* y la necesidad de iniciar antibioticoterapia ante la sospecha de infección de cualquier localización, se removió la indicación de antibioticoterapia cuando ocurre la ruptura prolongada de membranas (>18 h) puesto que esta consideración debe realizarse desde el ingreso, y si ingresara la paciente a la institución en expulsivo, tampoco tiene cabida en este apartado puesto que ante esta situación, deben ser llenados en este instante ambos momentos de la lista de chequeo, tanto el apartado correspondiente al ingreso (en donde esta indicación sí está referida) como el apartado justo antes del parto o la cesárea.

En el tercer momento, después del parto o la cesárea (y hasta una hora después), también se pregunta si la madre manejo su embarazo con sulfato de magnesio, y se le pide al clínico considerar las mismas indicaciones expuestas en los dos apartados anteriores. Allí también se hace referencia al uso de antibioticoterapia, ampliando las indicaciones de la lista original al agregar su uso ante el parto instrumentado, extracción manual de la placenta, revisión uterina, desgarro perineal severo y sospecha clínica de infección de cualquier localización. En este tercer momento aparecen las indicaciones referentes al bebé, y aquí, se mantienen las indicaciones de necesidad de remisión, antibioticoterapia y requerimiento de monitoreo especial haciendo modificaciones al ítem de antibioticoterapia, al descartar las convulsiones neonatales en esta etapa como indicación para administrar antibioticoterapia por considerar que este síntoma, justo al nacimiento, se debe a otras situaciones clínicas diferentes a infección neonatal, tales como trastornos hidroelectrolíticos, hipoglucemia y desordenes del metabolismo. Se adicionó la indicación de antibioticoterapia ante sífilis congénita y APGAR <7 (que predispone a mayores infecciones en el neonato). Se formuló una nueva pregunta que se refiere al tamizaje para hipotiroidismo congénito y la toma de una muestra de sangre para la hemoclasificación en el neonato, situación que debe realizarse en el mismo parto, antes del alumbramiento, con la obtención de sangre del cordón umbilical, para evitarle al bebé una punción innecesaria y que la toma de la muestra luego de una hora lleva a falsos resultados ^28^. Definir si él bebe presenta hipotiroidismo, una patología que debe tratarse desde el nacimiento, so pena de alteraciones en el desarrollo donde se incluye la alteración del desarrollo neurológico en el bebé.

Referente al último momento de la lista de chequeo, antes de dar el alta tanto a la madre como al bebe, también se establecieron las situaciones en las cuales se debe pensar en antibióticos. Se propone iniciar antibioticoterapia a toda madre con temperatura superior o igual a 38 grados y sangrado vaginal fétido como en la guía original, pero se adicionó iniciar el tratamiento con antibióticos cuando hay signos de infección en el sitio quirúrgico, superficial y profundo y una sospecha clínica de infección en cualquier localización. En cuanto al bebé, se dejan las mismas indicaciones establecidas en la lista original; en este momento ante convulsiones neonatales, la probabilidad de que estas sean generadas por infección es suficientemente alta. En cuanto a la infección materna por VIH, se agregó la necesidad de un seguimiento por infectología tanto a la madre como al bebé para su control a largo plazo. Se aclararon los momentos en que se debe otorgar la cita de control postparto por la consulta externa, por lo que se recomienda una cita a los siete días si el parto fue de bajo riesgo y de 48 horas si hubo factores de riesgo. Por último, se agregó una verificación de si la madre y el bebé recibieron tratamiento para sífilis, si la prueba fue positiva y si se retiraron sondas y catéteres de haber sido utilizados. Esto para evitar que la paciente y su bebé egresen del hospital con posibles infecciones o con factores de riesgo que permitan desarrollar una infección.

## Conclusión

Se espera que la versión para Colombia de la Lista de Verificación del Parto Seguro sea una herramienta que ayude a las diferentes instituciones en salud a mejorar la atención a las maternas y neonatos. Apoyando con esta versión en el cumplimiento de los objetivos de desarrollo sostenible en nuestro país. Se espera que sea especialmente útil en las zonas distantes donde muchas de las prácticas seguras no se cumplen de forma sistemática. Con todo esto se espera que contribuya a disminuir la morbi-mortalidad materna y neonatal,

## Anexo 1 Panel de Expertos

Médicos Especialistas: Alejandro Antonio Bautista Charry. Médico Ginecobstetra. Director departamento obstetricia y ginecología, Universidad Nacional de Colombia. 26 años de experiencia. E-mail: aabautistac@unal.edu.co Alejandro León Upegui Saldarriaga. Médico anestesiólogo. Clínica Universitaria Bolivariana. 18 años de experiencia. E-mail: alus@une.net.co Ana María Guevara Zambrano. Médico Ginecobstetra. Clínica Universitaria Bolivariana, Hospital Pablo Tobón Uribe. 10 años de experiencia. E-mail: anitaguevara4@yahoo.es Catalina María Valencia González. Médico Ginecobstetra. Jefe de la Unidad Fetal de la Clínica Reina Sofía. 14 años de experiencia. E-mail: cvalencia@colsanitas.com Germán Parra Corredor. Médico anestesiólogo, Clínica Colsánitas. 34 años de experiencia. E- mail: germanparra2000@yahoo.com Jairo Enrique Guerrero Giraldo. Médico Ginecobstetra. Jefe del Departamento de Ginecología y Obstetricia del Centro Médico Imbanaco. 10 años de experiencia. E-mail: jairoenrique.guerrero@imbanaco.com.co Javier Hernando Eslava-Schmalbach. Médico anestesiólogo. Vice decano de investigaciones Universidad Nacional de Colombia. 23 años de experiencia. E-mal: jheslavas@unal.edu.co Jorge Andrés Rubio-Romero. Médico Ginecobstetra. Profesor titular de la Universidad Nacional de Colombia. 34 años de experiencia. E-mail: jarubior@unal.edu.co José Antonio Rojas Suarez. Médico anestesiólogo. Coordinador de la Unidad de Cuidados Intensivos, Gestión Salud de Cartagena. 10 años de experiencia. E-mail: joseantonio.rojas.suarez@gmail.com Juan Carlos Bocanegra. Médico anestesiólogo. Clínica Universitaria Colombia. 15 años de experiencia. E-mail: bocanegra67@yahoo.com Luz María Gómez. Médico anestesiólogo. Subdirectora científica S.C.A.R.E. 22 años de experiencia. E-mail: lm.gomez@scare.org.co Nicolás Ramós Rodríguez. Pediatra Neonatólogo. Director Programas de Especialización en Pediatría y Neonatología Universidad del Bosque, Presidente Sociedad Colombiana de Pediatría. 25 años de experiencia. E-mail: bosquepediatria@yahoo.com Mauricio Vasco. Médico anestesiólogo. Coordinador comité de anestesia obstétrica S.C.A.R.E. 15 años de experiencia. E-mail: machuchovasco@yahoo.com **Enfermeras:** Claudia Patricia Henao López. Enfermera, Clínica Universitaria Bolivariana. 10 años de experiencia. E-mai: claudia.henao@upb.edu.co Dolly Magnolia González Hoyos. Enfermera Jefe. Docente Universidad de Caldas. 34 años de experiencia. E-mail: dolly.gonzales@ucaldas.edu.co Laura Vanessa Osorio Berrio. Enfermera, Clínica Universitaria Bolivariana. 2 años de experiencia. E-mail: laurav.osorio@upb.edu.co Sandra Milena Raigosa Villa. Enfermera, Clínica Universitaria Bolivariana. 7 años de experiencia. E-mail: sandra.raigosa@upb.edu.co

## Anexo 2. Lista de Verificación del Parto Seguro - Versión Colombia

**Antes del parto:** / LISTA DE VERIFICACIÓN DEL PARTO SEGURO

1. En el Ingreso	
¿La madre necesita ser remitida? ❏ No ❏ Sí, se ha organizado	Verifique los criterios de remisión de su institución
¿Se inició el partograma? ❏ No: iniciará a los ≥4cm de dilatación ❏ Sí	Inicie el trazo cuando la dilatación cervical sea ≥4cm, ● Cada 30 minutos: registre la frecuencia cardiaca, contracciones y frecuencia cardiaca fetal. ● Cada 2 horas: registre la temperatura ● Cada 4 horas: registre la presión arterial
¿La madre es alérgica a algún medicamento? ❏ No ❏ Sí ¿Cuál(es)? _____________________________	
La madre requiere:	
¿Antibióticos? ❏ No ❏ Sí, suministrados	Suministre antibióticos a la madre si se presenta cualquiera de las siguientes: ● Temperatura ≥38ºC ● Flujo vaginal fétido ● Portadora desconocida o confirmada de Estreptococo Agalactiae ● Sospecha clínica de infección de cualquier localización
¿Sulfato de Magnesio? ❏ No ❏ Sí, suministrado	Suministre sulfato de magnesio a la madre si se presenta cualquiera de los siguientes: ● Presión arterial sistólica ≥140 o presión arterial diastólica ≥90, acompañado de cualquiera de los siguientes: Cefalea severa, alteraciones visuales como visión borrosa o fosfenos, dolor en hipocondrio derecho o epigástrico, vómito, hematuria, oliguria, trombocitopenia o sospecha de Síndrome Hellp. ● Presión arterial sistólica ≥160 o presión arterial diastólica ≥110 ● Eclampsia (Convulsiones) ● Edad gestacional ≤32 semanas con feto viable y trabajo de parto fase activa (neuroprotección fetal).
¿Antihipertensivo? ❏ No ❏ Sí, suministrado	Administre antihipertensivo según protocolo institucional si: ● La presión arterial sistólica es ≥160 o presión arterial diastólica es ≥ 110
¿Tratamiento para Sífilis? ❏ No, prueba negativa confirmada ❏ Sí, suministrado	Aplicar protocolo institucional. Si se desconoce el estado seguir protocolo institucional para realización de la prueba para sífilis y de acuerdo al resultado definir tratamiento
¿Antorretrovirales? ❏ No, VIH negativo confirmado ❏ Sí, suministrados	En caso de prueba de VIH positiva, administre antirretrovirales según protocolo institucional. Si se desconoce el estado, ordenar prueba rápida de VIH y de acuerdo al resultado definir manejo según protocolo institucional.
Suministros: ❏ Confirme la disponibilidad de suministros y el cumplimiento del protocolo para el lavado de manos. ❏ Confirme la disponibilidad y uso de guantes para cada examen vaginal.	Utilice guantes estériles en caso de membranas rotas
❏ Se alentó y se permite la presencia de un acompañante durante el trabajo parto. ❏ La gestante o acompañante reconocen los signos de alarma y solicitarán ayuda, en caso de: Sangrado, palidez, sudoración; dolor abdominal permanente, dolor de cabeza severo, problemas visuales o auditivos, problemas para orinar, sensación de pujo	
**Diligenciado por: __________________________________________________**	
Esta lista no pretende ser exhaustiva y no debe sustituir la historia clínica del paciente o el partograma. Se insta a realizar las modificaciones necesarias de la lista de verificación para que se adapte a las prácticas locales. Para obtener más información sobre el uso recomendado de la lista, por favor consulte la sección **“Safe Childbirth Checklist Manual”**, en: www.who.int/patientsafety	
**2. Justo antes del expulsivo (o antes de la cesárea)**	
**¿Se diligenció el partograma?** ❏ No ❏ Sí	Inicie el trazo cuando la dilatación cervical sea ≥4cm, ● Cada 30 minutos: registre la frecuencia cardiaca, contracciones y frecuencia cardiaca fetal. ● Cada 2 horas: registre la temperatura ● Cada 4 horas: registre la presión arterial
**La madre requiere:**	
¿Antibióticos? ❏ No ❏ Sí, suministrados	Suministre antibióticos a la madre si se presenta cualquiera de las siguientes: ● Temperatura ≥38ºC ● Flujo vaginal fétido ● Portadora desconocida o confirmada de Estreptococo Agalactiae ● Sospecha clínica de infección de cualquier localización ● Cesárea
¿Sulfato de Magnesio? ❏ No ❏ Sí, suministrado	Suministre sulfato de magnesio a la madre si se presenta cualquiera de los siguientes: ● Presión arterial sistólica ≥140 o presión arterial diastólica ≥90, acompañado de cualquiera de los siguientes: Cefalea severa, alteraciones visuales como visión borrosa o fosfenos, dolor en hipocondrio derecho o epigástrico, vómito, hematuria, oliguria, trombocitopenia o sospecha de Síndrome Hellp. ● Presión arterial sistólica ≥160 o presión arterial diastólica ≥110 ● Eclampsia (Convulsiones) ● Edad gestacional ≤32 semanas con feto viable y trabajo de parto fase activa (neuroprotección fetal).
¿Antihipertensivo? ❏ No ❏ Sí, suministrado	Administre antihipertensivo según protocolo institucional si: ● La presión arterial sistólica es ≥160 o presión arterial diastólica es ≥ 110
**Confirme si los suministros esenciales se encuentran disponibles para la atención del parto:**	
Para la madre ❏ Guantes estériles ❏ Antisépticos para el lavado de manos y preparación de la paciente según protocolo institucional ❏ Uterotónicos, de preferencia Oxitocina	Cuidados para la madre inmediatamente después del parto: ● Confirme el nacimiento de un solo bebé y no de un parto múltiple. ● Realice manejo activo del tercer estadío de acuerdo a protocolo institucional. ● Confirme que el útero está contraído.
Para el recién nacido ❏ Toalla limpia ❏ Bisturí o tijera estéril para cortar el cordón ❏ Ligadura de caucho, pinza plástica o cintas umbilicales estériles ❏ Fuente de calor ❏ Dispositivo de succión ❏ Bolsa-válvula-mascarilla	Cuidados inmediatos para el recién nacido: ● Secar al recién nacido y mantenerlo caliente. ● Si no respira: estimular y limpiar las vías respiratorias. ● Si después de eso continua sin respirar: - Sujetar y cortar el cordón - Ventilar con bolsa-válvula-mascarilla - Solicitar ayuda - Seguir protocolo institucional de reanimación neonatal
❏ En caso de ser necesario se dispone de un asistente (personal de salud/acompañante) para ayudar en la atención del parto	
Diligenciado por: __________________________________________________	
Esta lista no pretende ser exhaustiva y no debe sustituir la historia clínica del paciente o el partograma. Se insta a realizar las modificaciones necesarias de la lista de verificación para que se adapte a las prácticas locales. Para obtener más información sobre el uso recomendado de la lista, por favor consulte la sección **“Safe Childbirth Checklist Manual”**, en: www.who.int/patientsafety

**Después del parto:** / LISTA DE VERIFICACIÓN DEL PARTO SEGURO

3. Después del parto (dentro de la primera hora)	
¿El sangrado de la madre es anormal?	Si el sangrado es anormal:
❏ No	● Masajee el útero
❏ Sí, active protocolo de manejo de hemorragia obstétrica institucional	● Utilice más uterotónicos
	● Inicie reanimación con líquidos endovenosos y considere hemoderivados
	● Trate la causa: tono, trauma, tejido, trombina (Nemotecnia cuatro T’s), atonía uterina, retención de restos placentarios, desgarro vaginal, ruptura uterina, coagulopatía
**La madre requiere:**	
¿Antibióticos?	Suministre antibióticos a la madre si se presenta cualquiera de las siguientes:
❏ No	● Parto instrumentado
❏ Sí, suministrados	● Extracción manual de la placenta.
	● Revisión uterina
	● Desgarro perineal severo
	● Temperatura ≥38ºC
	● Flujo vaginal fétido
	● Sospecha clínica de infección de cualquier localización
¿Sulfato de Magnesio?	Suministre sulfato de magnesio a la madre si se presenta cualquiera de los siguientes:
❏ No	● Presión arterial sistólica ≥140 o presión arterial diastólica mayor o igual a 90, acompañado de cualquiera de los siguientes: Cefalea severa, alteraciones visuales como visión borrosa o fosfenos, dolor en hipocondrio derecho o epigástrico, vómito, hematuria, oliguria, trombocitopenia o sospecha de Síndrome Hellp.
❏ Sí, suministrados	● Presión arterial sistólica ≥160 o presión arterial diastólica ≥110
	● Eclampsia (Convulsiones)
¿Antihipertensivo?	
❏ No	Administre antihipertensivo según protocolo institucional si:
❏ Sí, suministrado	● La presión arterial sistólica es ≥160 o presión arterial diastólica es ≥ 110
**El recién nacido requiere:**	
¿Ser remitido?	
❏ No	
❏ Sí, remitido	
¿Antibióticos?	Revise los criterios de remisión de su institución de salud
❏ No	Suministrar antibióticos al recién nacido si se le ha dado a la gestante por infección obstétrica o si presenta:}
❏ Si, suministrados	● Frecuencia respiratoria >60/min o <30/min
	● Retracción torácica, estertores o convulsiones
	● Pobre respuesta al estimulo
	● Temperatura <35ºC (sin incremento después de brindarle calor), o temperatura ≥38ºC
	● Sospecha de sífilis congénita
¿Cuidado especial o monitoreo?	Brindar cuidados especiales y monitoreo si el recién nacido presenta cualquiera de los siguientes:
❏ No	● Edad gestacional <37 semanas
❏ Sí, se ha organizado	● Peso al nacer <2500 gramos
	● Requirió antibióticos
	● Requirió reanimación neonatal
¿Antirretrovirales?	En caso de prueba de VIH positiva administre antirretrovirales según protocolo institucional para el recién nacido (la profilaxis debe comenzar dentro de las 12 horas después del parto)
❏ No	
❏ Sí, se ha organizado	
Se realizó tamizaje para hipotiroidismo congénito y hemoclasificación?	Tome la muestra de sangre neonatal según protocolo institucional
❏No, tome muestra	
❏Sí	
❏**Se inició la lactancia y el contacto piel a piel** (Si tanto la madre como el recién nacido se encuentran en buen estado de salud)	
❏**La gestante o acompañante reconocen los signos de alarma y solicitarán ayuda, en caso de:**	
Para la madre: Sangrado, palidez, sudoración; dolor abdominal permanente, dolor de cabeza severo, problemas visuales o auditivos, problemas	
para orinar, sensación de pujo.	
Para el recién nacido: dificultad para respirar/respiración rápida, fiebre, piel fría, succión débil, somnolencia o difícil de despertar.	
**Diligenciado por: ____________________________________________________**	
Esta lista no pretende ser exhaustiva y no debe sustituir la historia clínica del paciente o el partograma. Se insta a realizar las modificaciones necesarias de la lista de verificación para que se adapte a las prácticas locales. Para obtener más información sobre el uso recomendado de la lista, por favor consulte la sección **“Safe Childbirth Checklist Manual”**, en: www.who.int/patientsafety	
**4. Antes de dar el alta**	
**¿El sangrado de la madre está controlado?**	Si el sangrado es anormal:
❏No: tratar y posponer el alta	● Masajee el útero
❏Sí	● Utilice más uterotónicos
	● Inicie reanimación con líquidos endovenosos y considere hemoderivados
	● Trate la causa: tono, trauma, tejido, trombina (Nemotecnia cuatro T’s), atonía uterina, retención de restos placentarios, desgarro vaginal, ruptura uterina, coagulopatía
**¿La madre requiere antibióticos?**	Suministre antibióticos a la madre si se presenta cualquiera de las siguientes:
❏No	● Temperatura ≥38ºC en las últimas 24 horas
❏Sí: suministrar y posponer el alta	● Sangrado genital fétido
	● Sospecha clínica de infección de cualquier localización
	● Signos de infección del sitio quirúrgico superficial o profundo (episiorrafia, herida quirúrgica, endiometritis, miometritis, etc.)
**¿El recién nacido requiere antibióticos?**	Suministrar antibióticos al recién nacido si se presenta:
❏No	● Frecuencia respiratoria >60/min o <30/min
❏Sí: suministre antibióticos, retrase el alta, brinde cuidado especial y monitoreo.	● Retracción torácica, estertores o convulsiones
	● Pobre respuesta al estímulo
	● Temperatura <35ºC (sin incremento después de brindarle calor), o temperatura ≥38ºC
	● Dejó de lactar bien
	● Enrojecimiento periumbilical o drena pus
**¿El recién nacido está lactando bien?**	
	
❏No: retrase el alta hasta que establezca buenas prácticas de lactancia materna	
❏Sí	
**En caso de que la madre sea VIH positivo, ¿tanto la madre como el recién nacido tienen antirretrovirales (ARV) para 6 semanas?**	Si la madre es VIH positivo:
❏Sí	● Asigne cita de control por infectología o programa de VIH en consulta externa a la madre y al recién nacido antes de un mes
❏No, suspender lactancia materna y aplicar protocolo institucional	
❏**La madre y recién nacido recibieron tratamiento según el resultado de la serología**	
❏**Se retiraron catéteres y sondas, si fueron empleados**	
❏**Se ofreció consejería de planificación familiar y se refirió para la administración del método seleccionado**	
❏**Se asignó una cita de control**	
❏**La madre o acompañante reconocen los signos de alarma y solicitarán ayuda, en caso de:**	
**Signos de alarma en**	
**La madre:**	**El recién nacido:**
● Sangrado	● Dificultad para respirar / respiración rápida
● Dolor abdominal severo	● Fiebre
● Dolor de cabeza fuerte	● Piel fría
● Alteración visual	● Succión débil
● Dificultad para respirar	● Somnoliento o difícil de despertar
● Fiebre o escalofríos	● Tinte amarillo en ojos, piel, palmas de las manos o plantas de los pies
● Dificultad para orinar	
**Diligenciado por____________________________________________________**	
Esta lista no pretende ser exhaustiva y no debe sustituir la historia clínica del paciente o el partograma. Se insta a realizar las modificaciones necesarias de la lista de verificación para que se adapte a las prácticas locales. Para obtener más información sobre el uso recomendado de la lista, por favor consulte la sección **“Safe Childbirth Checklist Manual”**, en: www.who.int/patientsafety.
